# Synthesis and perceptual scaling of high-resolution naturalistic images using Stable Diffusion

**DOI:** 10.3758/s13428-025-02889-8

**Published:** 2025-12-10

**Authors:** Leonardo Pettini, Carsten Bogler, Christian Doeller, John-Dylan Haynes

**Affiliations:** 1 Bernstein Center for Computational Neuroscience Berlin, Charité Universitätsmedizin Berlin, corporate member of the Freie Universität Berlin, Humboldt-Universität zu Berlin, and Berlin Institute of Health, Berlin, Germany; 2 Max Planck Institute for Human Cognitive and Brain Sciences, Leipzig, Germany; 3 Max Planck School of Cognition, Leipzig, Germany; 4 Department of Psychology, Humboldt-Universität zu Berlin, Berlin, Germany; 5 Kavli Institute for Systems Neuroscience, Centre for Neural Computation, The Egil and Pauline Braathen and Fred Kavli Centre for Cortical Microcircuits, Jebsen Centre for Alzheimer’s Disease, Norwegian University of Science and Technology, Trondheim, Norway; 6 Clinic of Neurology, Charité-Universitätsmedizin Berlin, Berlin, Germany; 7 Berlin Center for Advanced Neuroimaging, Charité-Universitätsmedizin Berlin, Berlin, Germany; 8 Research Cluster of Excellence “Science of Intelligence”, Technische Universität Berlin, Berlin, Germany; 9 Berlin School of Mind and Brain, Humboldt-Universität zu Berlin, Berlin, Germany

**Keywords:** Perceptual scaling, Naturalistic images, Stable Diffusion, Working memory

## Abstract

Naturalistic scenes are of key interest for visual perception, but controlling their perceptual and semantic properties is challenging. Previous work on naturalistic scenes has frequently focused on collections of discrete images with considerable physical differences between stimuli. However, it is often desirable to assess representations of naturalistic images that vary along a continuum. Traditionally, perceptually continuous variations of naturalistic stimuli have been obtained by morphing a source image into a target image. This produces transitions driven mainly by low-level physical features and can result in semantically ambiguous outcomes. More recently, generative adversarial networks (GANs) have been used to generate continuous perceptual variations within a stimulus category. Here, we extend and generalize this approach using a different machine learning approach, a text-to-image diffusion model (Stable Diffusion XL), to generate a freely customizable stimulus set of photorealistic images that are characterized by gradual transitions, with each image representing a unique exemplar within a prompted category. We demonstrate the approach by generating a set of 108 object scenes from six categories. For each object scene, we generate ten variants that are ordered along a perceptual continuum. This ordering was first estimated using a machine learning model of perceptual similarity (LPIPS) and then subsequently validated with a large online sample of human participants. In a subsequent experiment, we show that this ordering is also predictive of stimulus confusability in a working memory task. Our image set is suited for studies investigating the graded encoding of naturalistic stimuli in visual perception, attention, and memory.

## Introduction

Naturalistic stimuli are important for understanding object recognition and memory in ecologically valid settings (Henderson & Hollingworth, 1999; Bar, 2004; Oliva & Torralba, 2007; Epstein & Baker, 2019), but they present several challenges. They can vary widely in their semantic and perceptual dimensions, which makes them harder to select and to control experimentally in comparison to low-dimensional stimuli (Goetschalckx et al., 2021). In contrast to traditional stimulus sets, which have relied on the manual selection of a limited set of cropped images (Snodgrass & Vanderwart, 1980; Brodeur et al., 2010), a process that is both time-consuming and susceptible to subjectivity biases, recent attempts have strived to systematically collect and use naturalistic stimuli. These approaches are usually “bottom-up”, involving the collection and categorization of large numbers of images from the internet (“scraping”). These can be found in large-scale image datasets used in the machine learning community for computer vision tasks, such as ImageNet (Deng et al., 2009) or the Microsoft Common Objects in Context (COCO) dataset (Lin et al., 2014). For example, the Natural Scenes Dataset (NSD) initiative (Allen et al., 2022) collected a rich amount of behavioral and neuroimaging data while participants viewed scenes from the COCO dataset. Such stimuli, however, are not always ideally suited for cognitive tasks and often require manual pre-selection. To address this issue, the THINGS initiative (Hebart et al., 2019) has developed a procedure to sample and evaluate a vast variety of “object concepts” from the web, specifically tailored for cognitive scientific tasks. Despite these advancements, these methods remain fundamentally dependent on existing images and their *post hoc* categorization.

An alternative approach involves synthesizing stimuli using modern machine learning technology. Generative models such as variational autoencoders (VAEs) (Kingma & Welling, 2022), generative adversarial networks (GANs) (Goodfellow et al., 2014) and diffusion models (DMs) (Ho et al., 2020) have already provided novel methodological approaches to many areas of neuroscience, including image reconstruction from neuroimaging data (Shen et al., 2019; Ozcelik & VanRullen, 2023; Liu et al., 2024), analysis of neural population dynamics (Pandarinath et al., 2018; Bashivan et al., 2019), and clinical imaging (Yi et al., 2019; Pinaya et al., 2023). They are also promising for the synthetic generation of naturalistic stimuli for experimental tasks (Goetschalckx et al., 2021; Son et al., 2022; Cooper et al., 2023). Compared to “bottom-up” approaches that rely on scraping pre-existing images from the web, generative models allow for the synthesis of virtually unlimited high-resolution images, removing the dependency on existing sources. This is particularly appealing for object recognition and memory research, as it enables the creation of a variety of objects from a wide range of categories.

One particularly interesting feature of artificially generated images is that they can potentially help tackle the trade-off between ecological validity and parametric experimental control. For example, low-level physical properties (such as orientation or luminance) can easily be gradually varied and they are thus suitable for psychophysical studies that involve assessment of quantitative properties (Schurgin et al., 2020). In contrast, sets of discrete naturalistic images do not exhibit the same kind of gradual local ordering that would be necessary for such quantitative assessments. Any measurement that requires a gradual variation of an image property is very difficult to realize with sets of pre-existing, discrete naturalistic stimuli.

Controlling perceptual and semantic features of high-dimensional naturalistic stimuli is inherently difficult. However, machine-learning-based approaches provide a solution. One class of machine learning methods, generative adversarial networks (GANs) (Goodfellow et al., 2014), has recently gained attention in cognitive science (Goetschalckx et al., 2021). The deep generative representations that GANs learn have been shown to be structured semantically (Yang et al., 2020). They allow obtaining fine-grained and relatively selective variations of the images along continuous dimensions, both for perceptual (e.g., the lightness of the scene) and for semantic features (e.g., facial attributes) (Shocher et al., 2020; Yang et al., 2021). A recent study, Son et al. (2022), used a GAN to create “scene wheels” of naturalistic indoor scenes with varying levels of similarity, which they employed in a visual working memory task, typically used for simpler features like color or orientation (Zhang et al., 2008). This reflects a growing interest towards a more ecologically valid assessment of cognitive function and provides the possibility to extend working memory models to naturalistic stimuli (Bates et al.., 2024). However, despite their utility, GANs face significant limitations. Pre-trained GAN models, while available (Karras et al., 2019), often require fine-tuning to be suitable for specific image generation tasks. Some of these models can generate a variety of images, but are restricted to certain categories, and their resolution can be rather low.

**Fig. 1:**
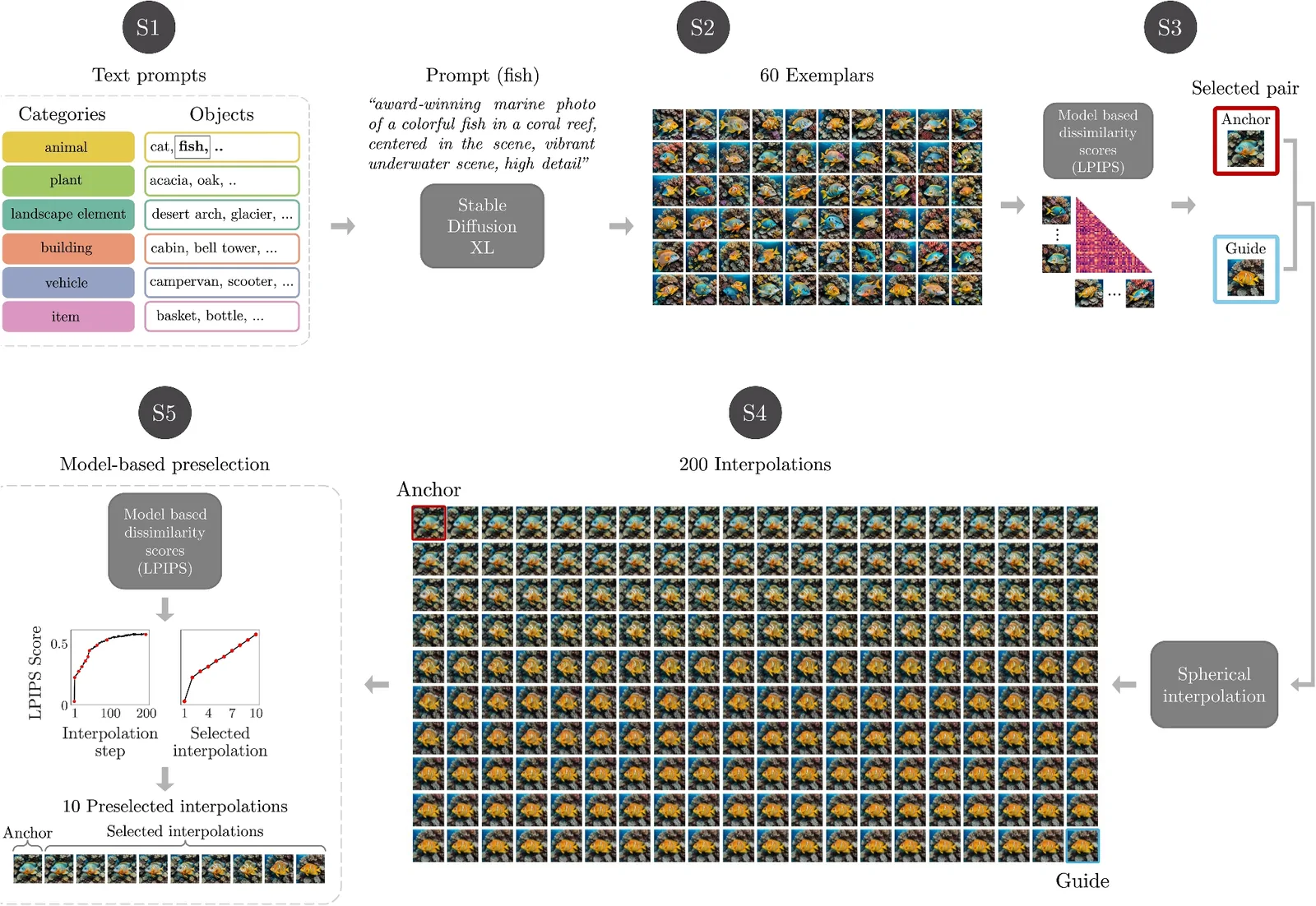
Overview of the pipeline to generate the stimulus set (Stage 1). We defined six global categories (three natural and three artificial), each including eighteen object scenes. To generate varied exemplar images for each object (shown here for “fish”), we used a single prompt, and variability was induced by using sixty starting noise latents (see Methods). From each set of exemplar images, we selected an anchor and a guide image for the interpolation, which was performed between their respective noise latents. Out of 200 interpolated images, we pre-selected ten (the anchor image and nine interpolations) using a computational model-based metric (LPIPS; Zhang et al., 2018) as an initial approximation to quantify their perceptual similarity

Another class of generative neural networks specialized in image synthesis is diffusion models (Ho et al., 2020; Nichol & Dhariwal, 2021; Song et al., 2022), which so far have received less attention in cognitive science. Stable Diffusion (Rombach et al., 2022), in particular, is an open-source model that can generate images from text prompts. Text-to-image approaches for stimulus generation are more flexible (Dhariwal & Nichol, 2021) because they allow a high flexibility in the choice of visual scenes without requiring scraping of sets of dedicated training samples.

In this study, we utilized the flexibility of diffusion-based text-to-image models to generate a novel set of naturalistic stimuli. The terms “natural” and “naturalistic” are often used interchangeably in cognitive science (Hebart et al., 2019, 2020; Gong et al., 2023). In order to avoid confusion with the machine learning terminology, which may consider the term “natural” as an antonym of “synthetic”, that is the “ground truth” images that generative models aim to replicate (Goodfellow et al., 2014; Dzanic et al., 2020), here we solely use the term “naturalistic” to describe synthetically generated, realistic-appearing images. We continue to use the term “natural” as opposed to “artificial” to denote the natural categories of stimuli (animals, plants, landscape elements). Note that, while approximately photorealistic, our images do not fully resemble real-world natural scenes (see Discussion for more on this point). We generated sets of “object-scenes” that have a central, prominent object situated in a coherent scene, in line with previous work (Hebart et al., 2019). To generate these stimuli, we used Stable Diffusion XL (Podell et al., 2023), a diffusion model that excels in the synthesis of high-resolution images (1024 x 1024 pixels). We also ensured and assessed the psychometric continuity in a three-step procedure. First, we ordered images using a machine-learning-based psychophysical similarity metric, learned perceptual image patch similarity (LPIPS) (Zhang et al., 2018). Then, in a second step, we fine-tuned the ordering of stimuli using an online similarity judgement with a sample of 1113 human participants. In a third step, we assessed whether perceptual similarity generalizes to other cognitive functions, specifically to working memory representations. For this, we used our novel stimulus set in a visual working memory task. Specifically, this experiment assessed visual working memory performance for stimuli at varying levels of distance in our continuous stimulus sets.

The results confirmed that our stimuli effectively captured perceptual variations, making them a useful resource for studying memory and perception under controlled yet ecologically valid conditions. By providing the stimulus set publicly (see Data availability section), we hope to contribute a valuable tool for the research community, bridging the gap between ecological validity and experimental control in visual cognition studies.

## Methods

The study consisted of three main parts: In Stage 1 ("Stimulus set generation"), we used a diffusion model (Ho et al., 2020; Nichol & Dhariwal, 2021; Song et al., 2022), specifically Stable Diffusion XL (Podell et al., 2023), to generate large sets of exemplars of object scenes, each described by a text prompt (e.g., for "fish", we used *"award-winning marine photo of a colorful fish in a coral reef, centered in the scene, vibrant underwater scene, high detail"*). We then estimated the perceived similarity of the different exemplars based on a computational model (learned perceptual image patch similarity, LPIPS) (Zhang et al., 2018) and ordered the scenes accordingly. In Stage 2 ("Perceptual similarity judgement experiment"), we validated and subsequently fine-tuned the perceptual ordering by performing a similarity judgement task with an online sample of 1113 participants. In Stage 3 ("Memory validation experiment"), we additionally validated that the proximity along our perceptual continua predicted performance in a separate working memory task.

### Stage 1: Image generation using the diffusion model

The image generation proceeded in five steps (shown in Fig. 1; for full details see the Appendix): (S1) We used Stable Diffusion XL in combination with text prompts to generate naturalistic images ("object scenes") with a clear, central object situated in a coherent scene, rich in detail (see Appendix A.1.1 for full specifications). Since text-to-image models (Zhang et al., 2023) can generate multiple images from a textual prompt, there are potentially infinite images that can be synthesized. We therefore narrowed down the prompt space by defining six different categories from which our objects were taken. These categories were split into natural (animals, plants, landscape elements) and artificial (vehicles, items, buildings). For each category, we identified 18 unique object scenes that were distinct yet representative of the category (e.g., "fish" and "beaver" were two object scenes of the category "animals"). Each of the 108 object scenes had an associated unique text prompt. See Appendix A.1.2 for details about the prompt selection. (S2) Based on these text prompts, we used Stable Diffusion XL (Podell et al., 2023) to generate 60 exemplar images per each of the 108 object scenes. These were different realizations of the same object scene (e.g., 60 different realizations of the object scene "fish"; see Fig. 1 and Appendix A.1.2). (S3) For each pair of these 60 exemplar images from one object scene, we computed a coarse-grained model-based perceptual similarity score using an established metric based on neural network activation patches, the learned perceptual image patch similarity (LPIPS) metric (Zhang et al., 2018). From this, we selected an “anchor image” and a “guide image”. These two images were chosen such that they were representative of the set as well as perceptually similar to one another (for details see Appendix A.1.3). (S4) We then used spherical interpolation to yield 200 further images per object scene on the continuum between the anchor image and the guide image (this two-step procedure was done in order to go from coarse-grained to fine-grained steps). This interpolation was done at the level of noise latents rather than at the semantic level of the text prompts in order to ensure that interpolated images varied perceptually but not in terms of meaning (see Appendix A.1.4). (S5) We used the artificial neural network model (LPIPS) once more to compute the fine-grained model-based perceptual similarity between these 200 interpolations. Based on this, we selected a total of ten images that were dissimilar from the anchor image in an approximately linear way (see Appendix A.1.5).

### Stage 2: Psychophysical similarity judgement task

#### Participants

We recruited 1285 participants on the online platform Prolific for an experiment involving a perceptual similarity judgement task. This sample was larger than the target minimum (at least 20 participants judging an object, for a total of 1080) because we anticipated excluding up to 20% of the data, as reported previously (Gagné & Franzen, 2023; Uittenhove et al., 2023). The final sample included 1113 participants (age 18–40, M=28.9, SD=5.7; 760 male, 521 female, and five "prefer not to say"). Participants received remuneration of 8.53 GBP (approximately 10 EUR) per hour. They were selected from a standard sample (aged 18–40 years, fluent in English) to maximize data quality. We excluded participants who did not engage properly with the task or experienced technical problems (see exclusion criteria in the Appendix A.2.4). All participants provided informed consent before taking part in the study. The consent process included detailed information about the purpose and duration of the experiment, voluntariness of participation, and data protection policies. The study was approved by the Ethics Committee of the Institute of Psychology of the Humboldt University of Berlin, Germany.

#### Experimental procedures and design

Participants performed a similarity judgement task based on triplet comparisons, or “method of triads” (Torgerson, 1952), which is used both in psychophysics and machine learning (Aguilar et al., 2017; Demiralp et al., 2014; Haghiri et al., 2020; Künstle et al., 2022; Li et al., 2016; Wichmann et al., 2017). Given a set of stimuli $S = \{s_1, s_2, \ldots , s_n\}$, where n is the total number of stimuli, participants are asked to judge the similarity of three stimuli at a time. In particular, given a triplet of stimuli $(s_i, s_j, s_k)$, they are asked which between two probe stimuli $s_j$ and $s_k$ is most similar to a reference stimulus $s_i$. After providing informed consent, participants received detailed instructions on the task procedure and had to pass an attention check to make sure they understood the instructions. Before starting the main task, they underwent a training session where they performed 12 trials using an independent set of stimuli. The main task consisted of 288 trials divided in six blocks of 48 trials each. In between blocks, participants were prompted to take a short break (maximum 2–3 minutes) to maintain attention and accuracy. Each trial began with a fixation target (Thaler et al., 2013) presented at the center of the screen for a jittered duration between 500 and 1000 ms, which was randomized in 100-ms steps. Then, participants were shown three images: one reference image on top and two probe images below (see Fig. 8). Their task was to judge which of the two probe images was more similar to the reference image. Full details of the experimental procedures can be found in the Appendix.

#### Analyses

The main goal of the similarity judgement analysis was to estimate a perceptual scale for each object and its variations, given a set of perceptual judgements. To analyze the triplet judgements, we used three algorithms: maximum likelihood difference scaling (MLDS) (Maloney & Yang, 2003), which is a well-established scaling method in psychophysics, and two ordinal embedding algorithms, soft ordinal embedding (SOE) (Terada & Von Luxburg, 2014) and *t*-distributed stochastic triplet embedding (*t*-STE) (Van Der Maaten & Weinberger, 2012). MLDS can be used only in one-dimensional cases, whereas *t*-STE and SOE have been proposed to find ordinal embeddings in higher-dimensional spaces (Haghiri et al., 2020). Embeddings, in this context, are Euclidean representations that preserve the ordinal relationships among data points based on a set of perceptual triplet judgements (Agarwal et al., 2007; Jamieson & Nowak, 2011). We performed a comparative analysis between the three algorithms, assessing both their performance and the stability of the embedding estimates. To assess their performance, we used the cross-validated triplet error $E_t$ (Haghiri et al., 2020).

For each object and category, we calculated the mean cross-validated triplet error of each algorithm by averaging the triplet errors from all cross-validation steps. The object-wise and category-wise errors estimated how well the model performed for individual objects and for categories. We also calculated the overall mean triplet error for each algorithm, which provided a general measure of how they performed across objects. As a final step, we reordered the images according to the embedding results and compared this order to the one defined by the LPIPS metric.

### Stage 3: Memory validation task

#### Participants

For the second experiment, we recruited 338 participants from the online platform Prolific using the same criteria as in the Psychophysical Similarity Judgement Task. The final sample included 240 participants (age 18–40, M=28.42, SD=5.86; 138 male, 101 female, and one "prefers not to say"). We excluded participants who did not engage properly with the task, who showed behavioral performance below chance, and who reported technical problems (for detailed criteria see Appendix A.3.1). The study was approved by the Ethics Committee of the Institute of Psychology of the Humboldt University of Berlin, Germany.

#### Stimuli

From the ten-image set for each of the 108 object scenes, we selected a target image and three foils with increasing perceptual dissimilarity. Since our final image sets contained ten images per object, we implemented an algorithm to subsample four images using their embedding values and avoiding abrupt perceptual changes as much as possible (see Appendix A.3.2).

#### Experimental procedures and design

Participants began by reviewing and signing an online consent form, after which they received detailed instructions about the experimental procedure. They then completed a brief training session within their browser to familiarize themselves with the task. At the end of the experiment, they were asked to fill in a short debriefing questionnaire. In line with previous work (Daniel et al., 2016), we used a delayed match-to-sample paradigm with repeated and non-repeated target images (Fig. 6). The experiment was divided into six blocks of 18 trials each, for a total of 108 trials. At the beginning of each trial, a target image, which participants were instructed to memorize, was shown in the center of the screen for 2 s, followed by an 8-s delay with a blank screen. Then, participants were tested on whether they could recognize the same target image among a perceptually similar foil chosen from our novel stimulus set. We selected three foils with increasing levels of dissimilarity (see below for details) to manipulate task difficulty across three levels: easy, medium, and hard. The target and foil were sequentially presented for 1 s each, separated by a 500-ms gap, and their order was counterbalanced so that sometimes the target was shown first and sometimes second. Once both images had appeared, a new response mapping screen was presented with two options displayed in a monospaced font to the left and right of a question mark, respectively, with their positions on the screen counterbalanced (see below). Participants used this screen to indicate which image was the target by selecting either “one” or “two,” corresponding to pressing the ‘f’ key if the chosen option appeared on the left, and the ‘j’ key if it appeared on the right. The response window had a fixed duration of two seconds, regardless of when they pressed a key. Upon key press, participants were given visual feedback indicating the selected option (but not whether the response was correct). An inter-trial interval was randomly selected from 1000 ms, 1500 ms, and 2000 ms to introduce temporal variability and reduce predictability. Participants were instructed to maintain stable fixation throughout the task by fixating on a central target (a bull’s-eye with crosshairs) that remained visible at the center of the screen, even during stimulus presentation (Thaler et al., 2013). The fixation target was also presented at the beginning and end of each block. The total trial duration was about 19 s.

Participants were assigned nine repeated targets and 54 non-repeated targets from the stimulus set, including 108 objects (see above). Each experimental block contained nine repeated targets and nine non-repeated targets. We ensured that repeated and non-repeated targets were systematically counterbalanced across participants. Each object appeared as a repeated target for an equal number of participants, while the distribution of non-repeated targets varied across the stimulus set, though the final count was similar. Within each block, difficulty levels were distributed evenly across repeated and non-repeated targets (i.e., three easy, three medium, and three hard trials for each category), so that the average difficulty of each block is constant. For each repeated target, we showed all difficulty levels in a random order before repeating them, which means that the overall object-specific difficulty was fully counterbalanced every three blocks. The presentation order of target and foil, as well as the left/right placement of the two response choices (“one” and “two”), was evenly split and randomized throughout the experiment so that the correct response occurred equally often on each button press. This constraint was designed to limit any systematic response bias.

#### Analyses

We calculated participants’ average accuracies over blocks for each target image condition (repeated vs. non-repeated) and difficulty level (easy, medium, hard). In order to additionally quantify learning effects over time for each experimental condition, we adopted a Bayesian framework. Bayesian methods offer several advantages for this type of analysis, including the ability to compute estimates and credible intervals for predicted quantities directly (Wagenmakers et al., 2018). Moreover, they facilitate the use of hierarchical structures to model individual differences in learning trajectories. We used a Bayesian hierarchical logistic regression model, which was implemented using the brms package (Bürkner, 2017) in R (R Core Team, 2024). The Bayesian model was estimated using Markov chain Monte Carlo (MCMC) sampling with four chains, each consisting of 10,000 iterations, including 2000 “warm-up” iterations. This resulted in a total of 32,000 post-warmup draws. The model was fitted to a total of 25,920 trials. Convergence was assessed using the potential scale reduction factor (PSRF), denoted as $\hat{R}$. The resolution of the chains was evaluated using the effective sample size (ESS) (Kruschke, 2021). The full specification of the model is provided in the Appendix A.3.3.

## Results

### Stage 1: Stimulus set generation

**Fig. 2:**
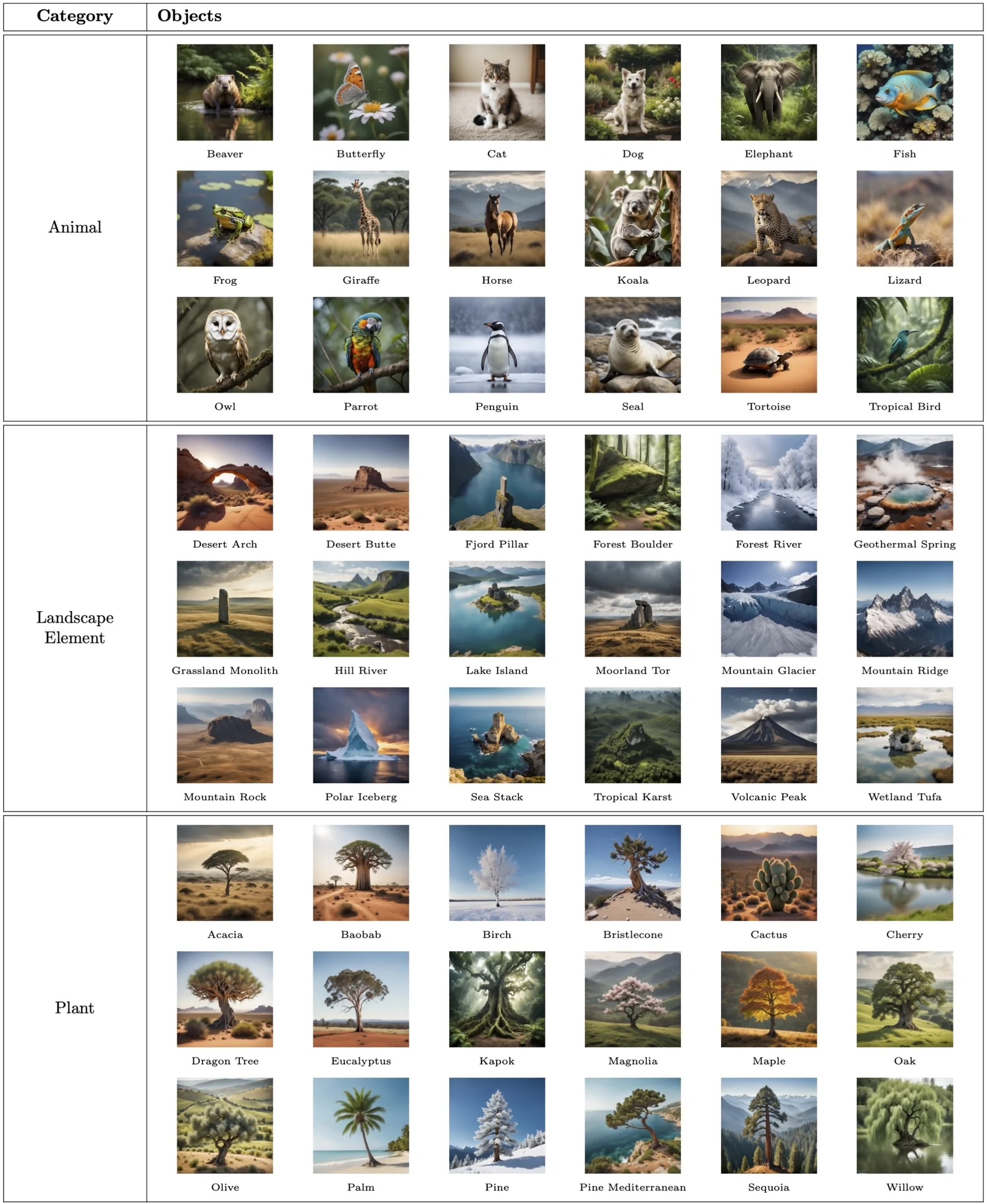
Table showing the categories and objects in the stimulus set. The categories in this table are classified as *natural*. For each object, the *anchor image* is shown

**Fig. 3:**
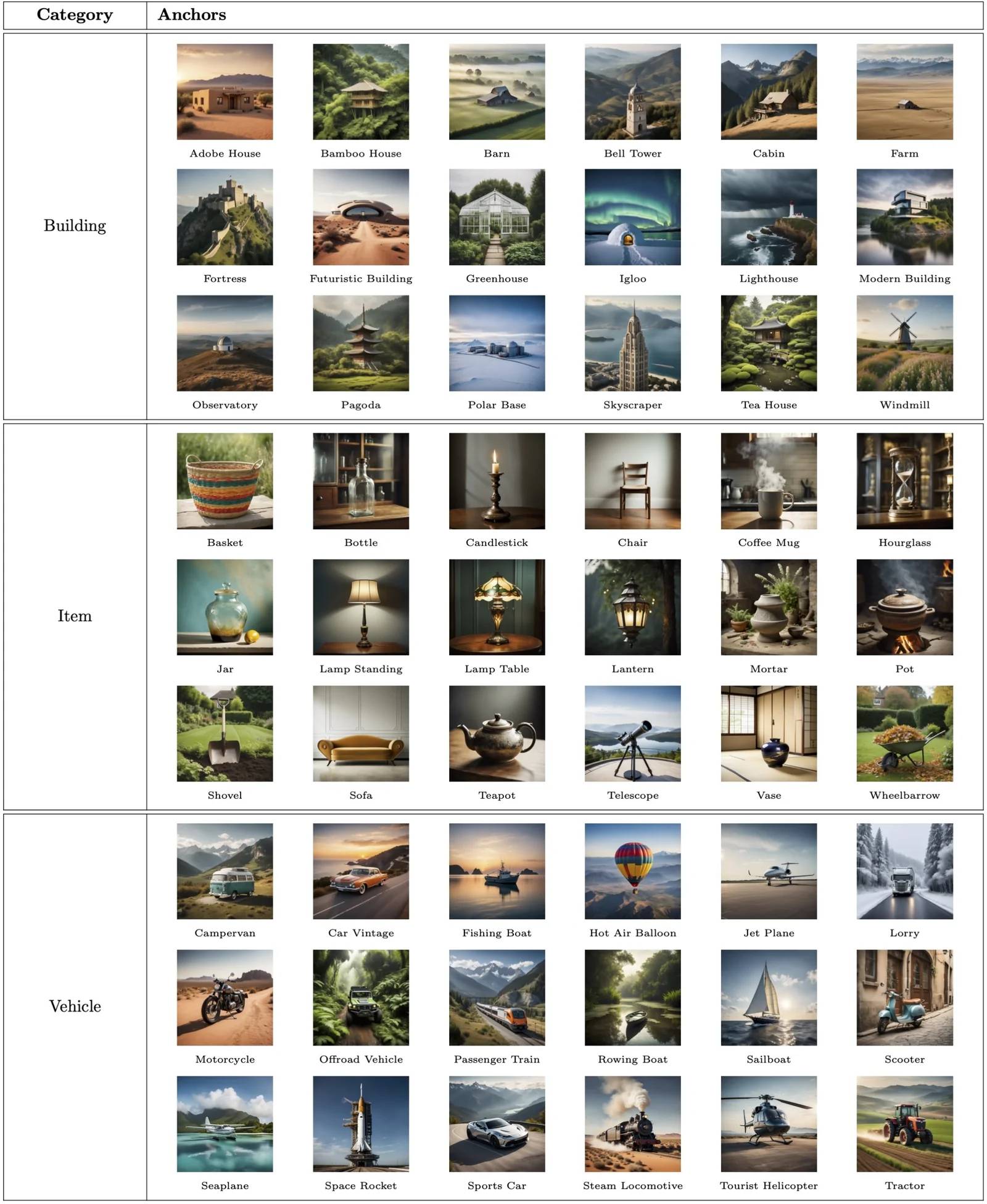
Table showing the categories and objects in the stimulus set. The categories in this table are classified as *artificial*. For each object, the *anchor image* is shown

In the first steps (S1-S2), our procedure generated 60 object scenes for 18 objects taken out of six categories (shown in Fig. 1 for the example object scene "fish"). After the next steps (S3-S5) our procedure generated an ordered set of ten images for each object scene based on the LPIPS, a machine learning model of perceptual similarity (Zhang et al., 2018). Specifically, each set comprised ten slightly different realizations of the same object (see Fig. 1, bottom left for "fish"). Within each object set, the images formed a perceptual continuum. Interpolation at the level of “noise latents” with a fixed text prompt successfully generated image sets where each image represented a distinct realization of the same semantic content (see Appendix B.1). Figure 2 shows examples for each of the 18 objects in the three natural categories, and Fig. 3 shows examples for each of the 18 objects in the three artificial categories. Out of the 60 exemplar images created for each object, we selected an anchor and a guide image (see Appendix A.1.3). Figures  2 and  3 show the anchor images that were used as a starting point for the subsequent interpolation.

As in previous studies (Cao et al., 2024), we implemented a systematic process to exclude images with artefacts, resulting in a 3.3% exclusion rate (Fig. 13). Generating smooth transitions was challenging, likely due to the non-linear nature of the high-dimensional space in which the interpolations were performed. Even though increasing the interpolation steps did make the transitions smoother, we occasionally observed perceptual “jumps” up to 1000 interpolation steps. Visual inspection revealed that most interpolated images were of high quality. A small proportion (12.4%) of images had to be replaced because they exhibited artefacts (see Appendix B.1; Fig. 16; Table 3). We also checked whether there were any overall differences in model-based similarity (LPIPS) scores across the different categories. We found that only *buildings* and *items* deviated in their scores (for full details, see Appendix B.1.2). Furthermore, we assessed physical properties of the anchor images and found they were similar across categories (Figs. 13(a) and 13(b) in Appendix B.1.4).

### Stage 2: Similarity judgement task

**Fig. 4:**
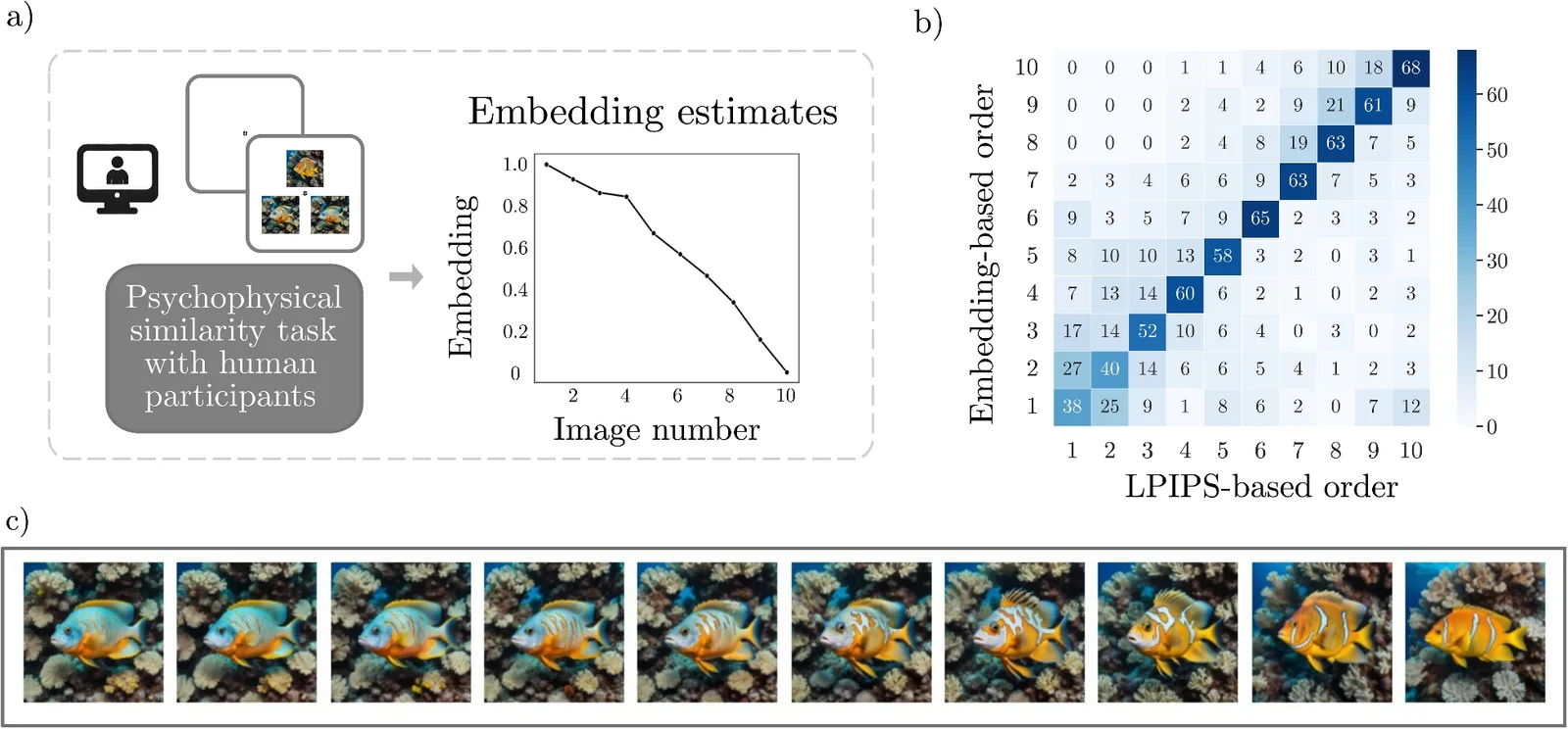
Psychophysical validation (Stage 2). **a** We conducted an online similarity judgement task using triplet comparisons to confirm and potentially fine-tune the order for each object set. The *x*-axis shows the ten images, and the *y*-axis shows the psychophysical embedding score. Relative distances on this embedding score correspond to perceptual differences. **b** Alignment between the LPIPS-based order and the embedding-based order derived from human similarity judgements. Each cell indicates the number of images assigned to the corresponding rank combination by both metrics. Higher counts along the diagonal indicate stronger agreement between LPIPS distances and perceived similarity. **c** Final image set ordered by embedding values. For the object "fish", LPIPS distances fully matched human similarity judgements

**Fig. 5:**
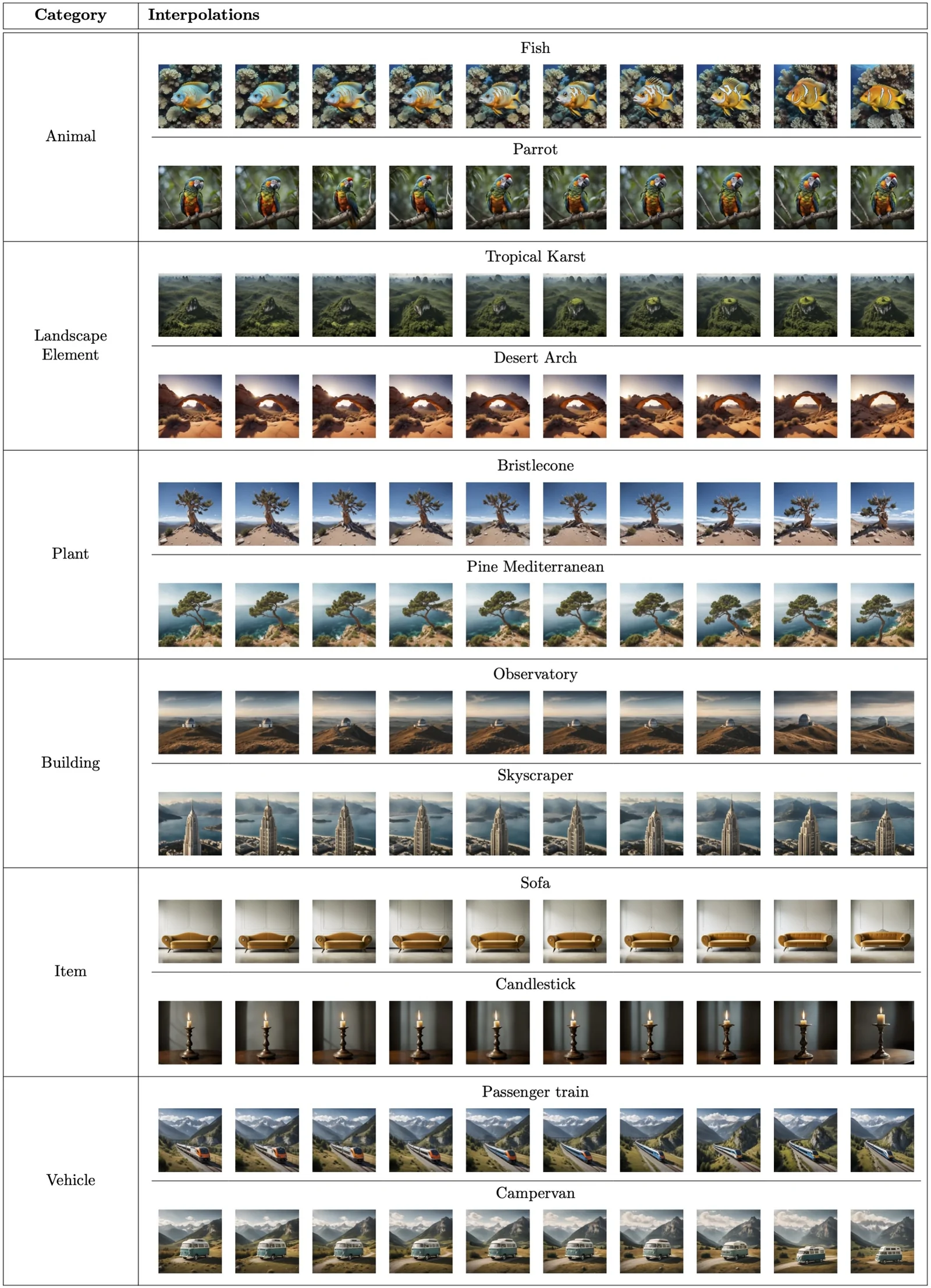
Examples of the final ordered image sets based on the psychophysical crowd-sourcing task. The figure shows the series for two out of 18 exemplars of each category. The anchor image is on the left

To validate the LPIPS-based ordering of each object set, we ran an online crowdsourced psychophysical similarity task using triplet comparisons (Fig. 4a, for details about the task cf. Appendix A.2). These judgements were used to estimate a one-dimensional embedding representing the perceived similarity scale within each object set. We assessed the degree to which the ordering derived from human judgements aligned with the order based on the LPIPS score. The matrix shown in Fig. 4b reports how often images occupied the same rank position within an object set across both methods. Overall, we observed strong alignment between the two orderings, with a Spearman rank correlation of ρ=0.73. Figure 4c shows the output of this stage for the example object scene ”fish”. Figure 5 displays the final image sets for two example objects from each of the 18 categories, ordered according to psychophysical judgements. Additional comparisons between the original LPIPS-based orderings and those adjusted using behavioral responses are shown in Fig. 24 (Appendix B.2.4).

### Stage 3: Memory task

**Fig. 6:**
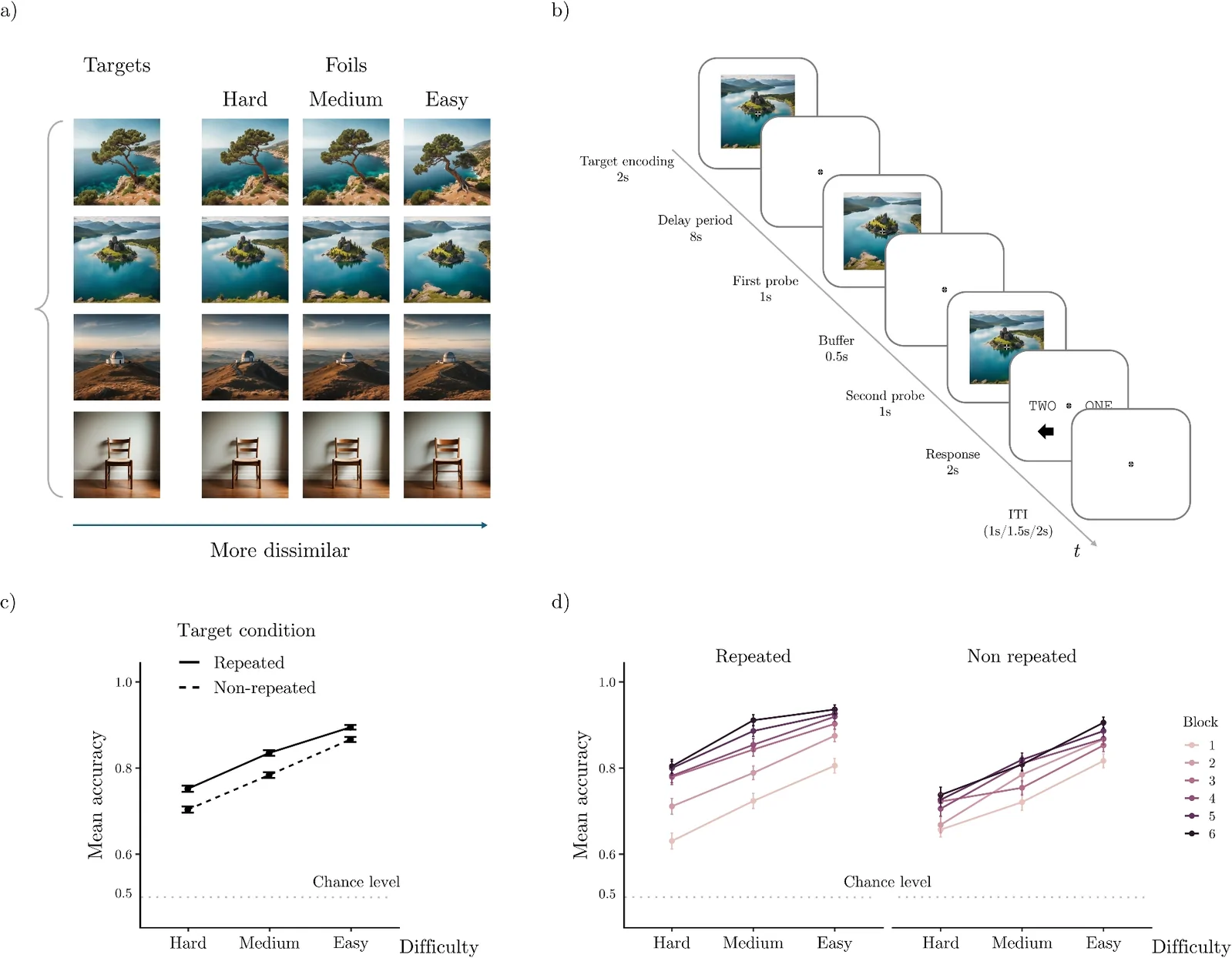
Results of the memory validation task (Stage 3). **a** Example image sets showing a target alongside three foils for four distinct object scenes (from top to bottom: Mediterranean pine, lake island, observatory, chair). **b** Schematic of a trial in the delayed match-to-sample paradigm. **c** Mean accuracy for repeated and non-repeated targets. **d** Mean accuracy across difficulty levels, grouped by block and target condition. Error bars indicate standard error of the mean (SEM)

**Fig. 8:**
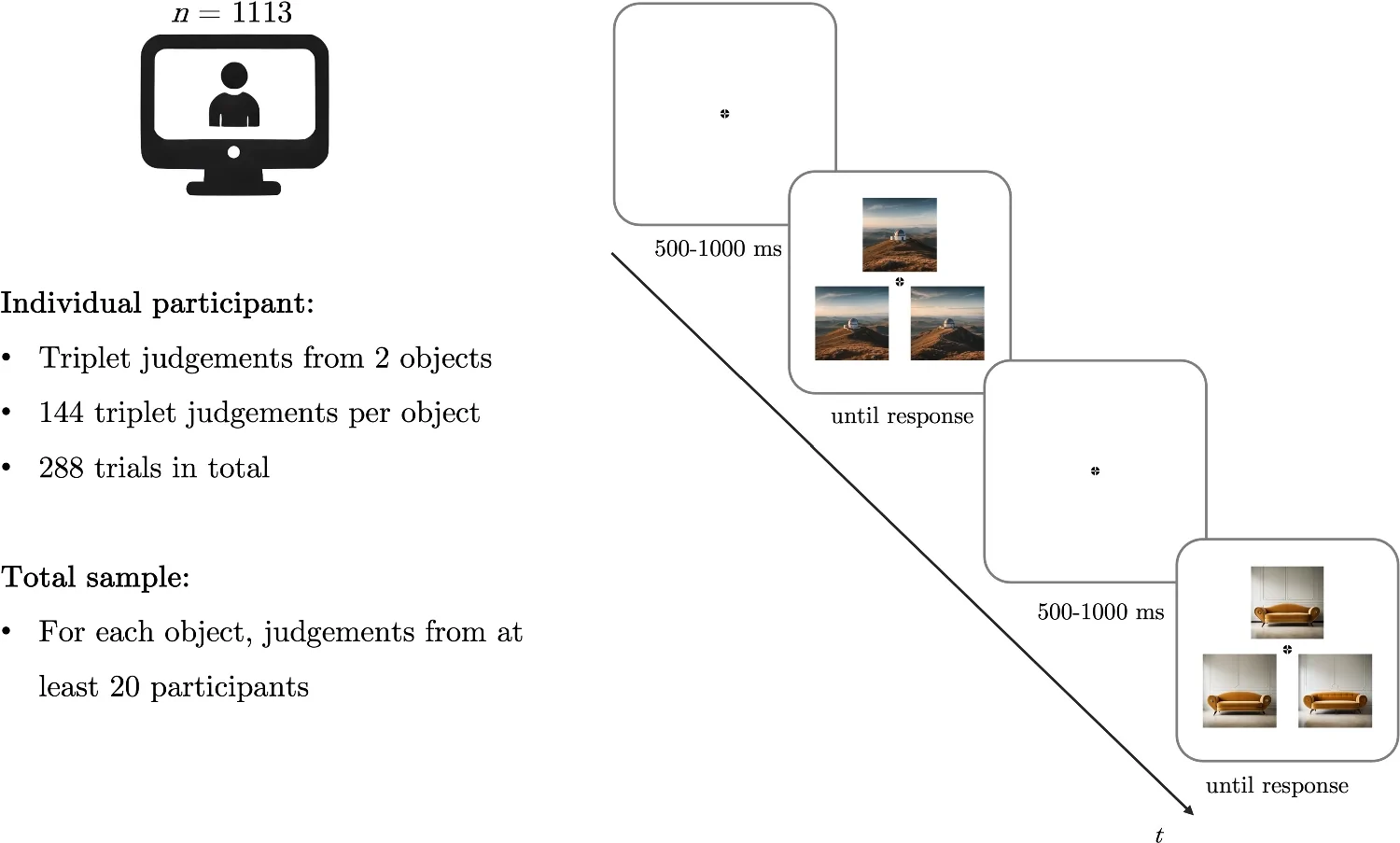
Online task design with two example trials of the similarity judgement task (for *observatory* and *sofa*). Participants were tasked to indicate which of the two bottom images is most similar to the top image. Each participant was assigned two objects and the triplet comparisons were always between variations of the same object

**Fig. 13:**
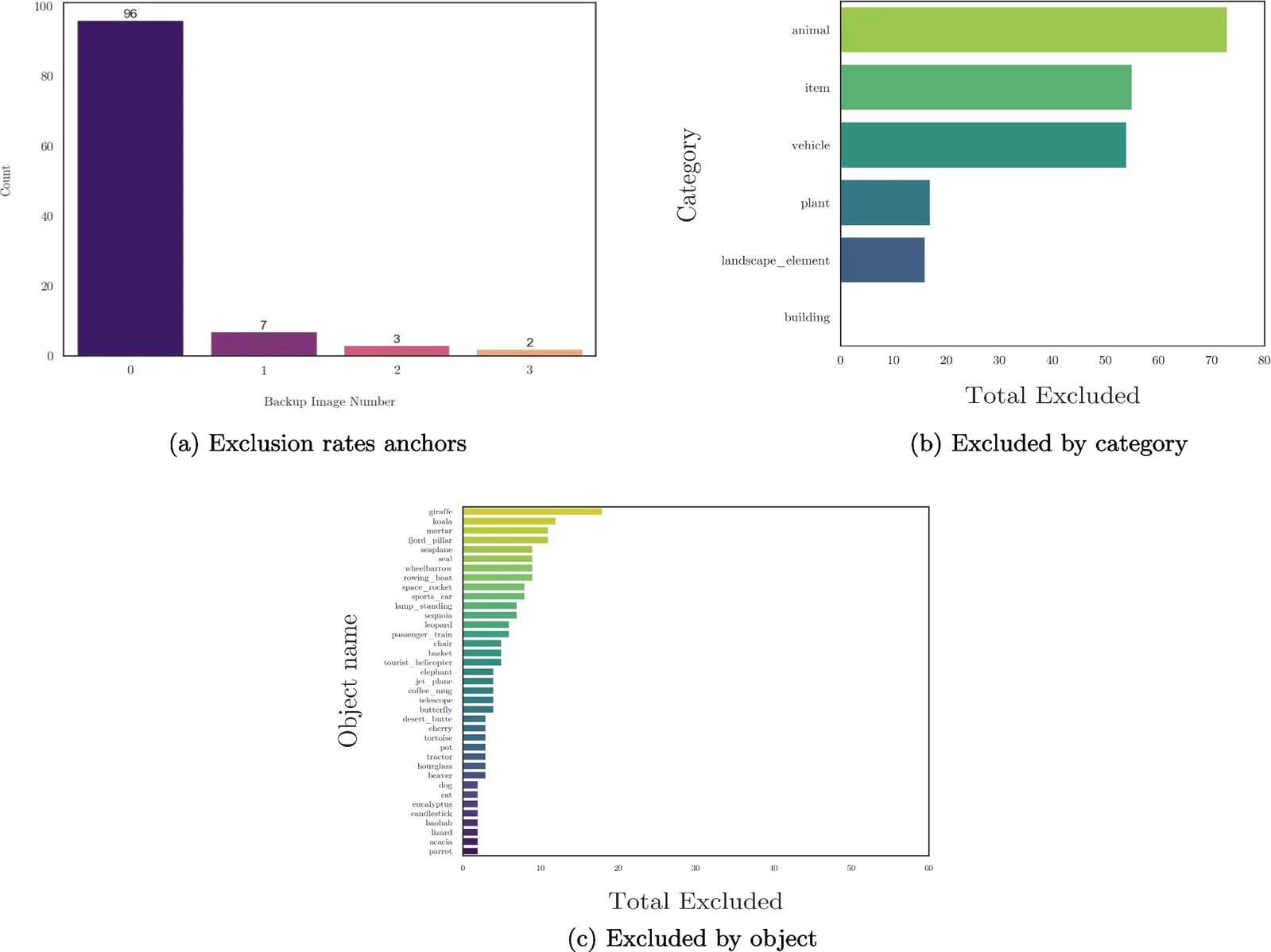
The giraffe had the highest number of exclusions, with 18 images excluded, which represents 30% of the 60 images for this object and 8.4% of the total 215 excluded images. This was followed by the koala with 12 exclusions (20% of its images and 5.6% of the total exclusions), and both the fjord pillar and mortar each with 11 exclusions (18.3% of their images and 5.1% of the total exclusions). The seal, seaplane, rowing boat, and wheelbarrow each had nine exclusions (15% of their images and 4.2% of the total exclusions). The space rocket and sports car each had eight exclusions (13.3% of their images and 3.7% of the total exclusions). Other notable exclusions included the lamp standing and leopard, each with seven exclusions (11.7% of their images and 3.3% of the total exclusions), and the passenger train with six exclusions (10% of its images and 2.8% of the total exclusions). Objects such as the tourist helicopter, elephant, coffee mug, butterfly, and telescope had between three and five exclusions (ranging from 5% to 8.3% of their images and 1.4% to 2.3% of the total exclusions)

**Fig. 16:**
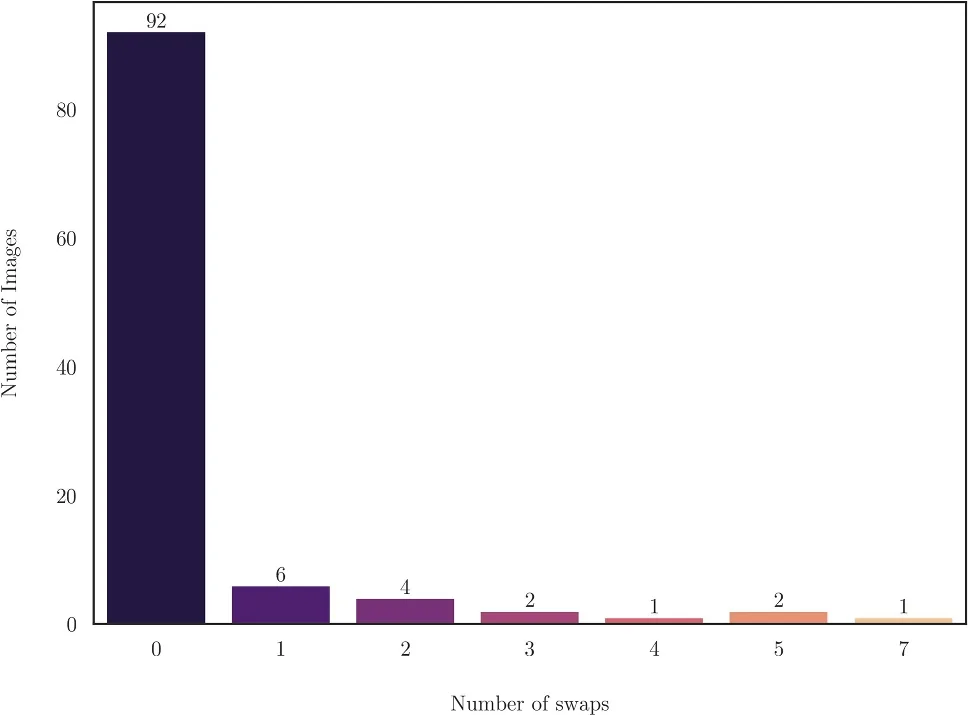
Swapped interpolations. Specifically, the following objects required swaps: *butterfly*, *horse*, *koala*, and *owl* each required 1 swap (10%). The mortar in the *item* category required 1 swap (10%), and the *table lamp* required 2 swaps (20%). The *fjord pillar* in *landscape elements* required 1 swap (10%). Among *animals*, the *cat*, *leopard*, and *parrot* each required 2 swaps (20%). For *vehicles*, the *passenger train* and *seaplane* required 3 swaps each (30%), and the *rowing boat* required 5 swaps (50%). The *teapot* required 4 swaps (40%), and the *telescope* required 5 swaps (50%) in the item category. The *giraffe* had the highest number of swaps, with 7 swaps (70%)

**Fig. 24:**
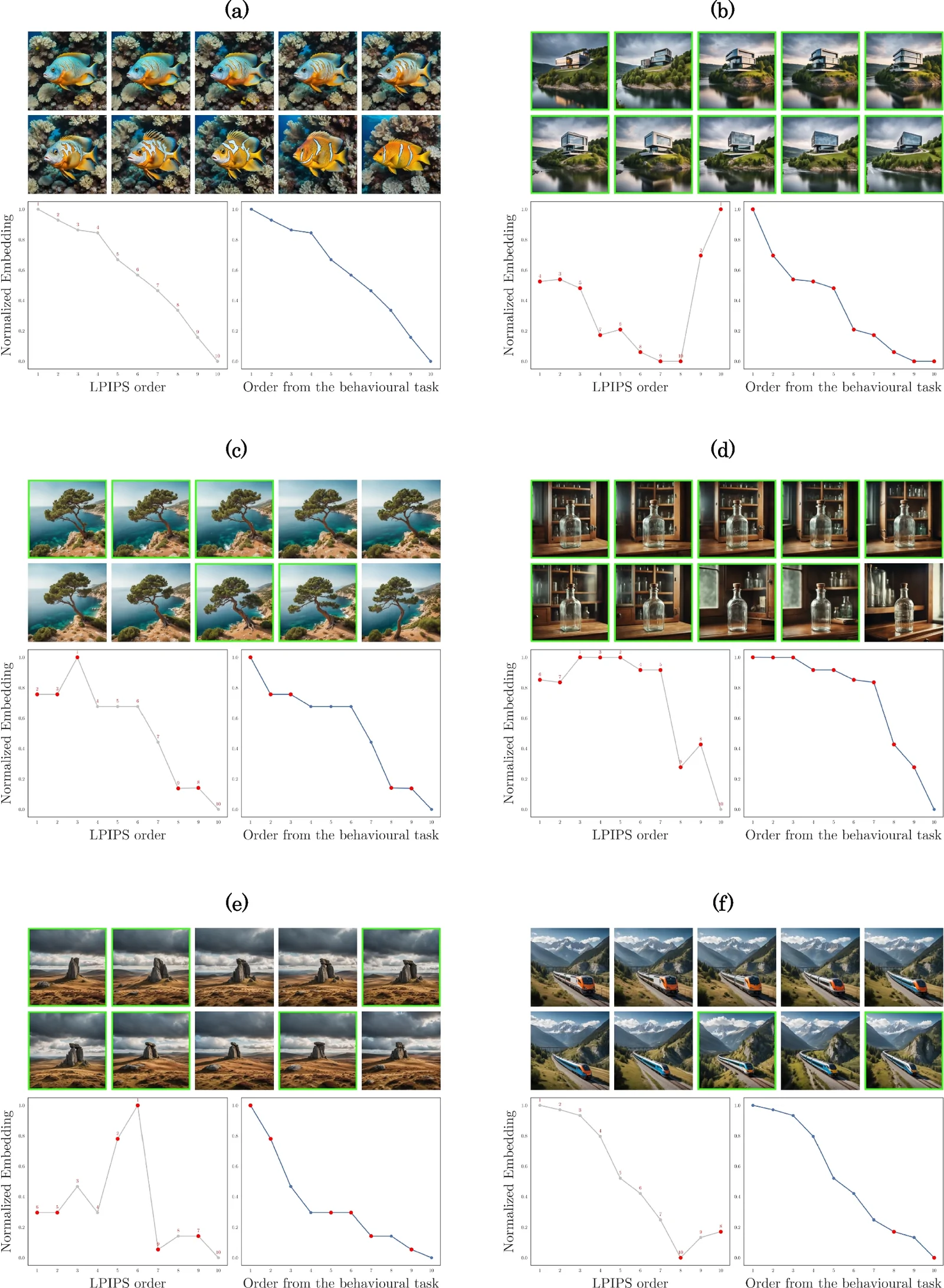
Example reorderings based on embedding values. Each panel displays images ordered by their embedding values. Images where the order was changed compared to the LPIPS model are highlighted in *green*. The *left plot* shows the original order, with red numbers indicating new positions post-reordering. The *left plot* displays the reordered objects sorted by embedding value. The *x*-axis represents object positions, while the *y*-axis shows embedding values. *Red dots* mark changed positions, highlighting adjustments in object order. Panels: **a** *fish*, **b** *modern building*, **c** *Pine (Mediterranean)*, **d** *bottle*, **e** *moorland tor*, **f** *passenger train*

**Fig. 25:**
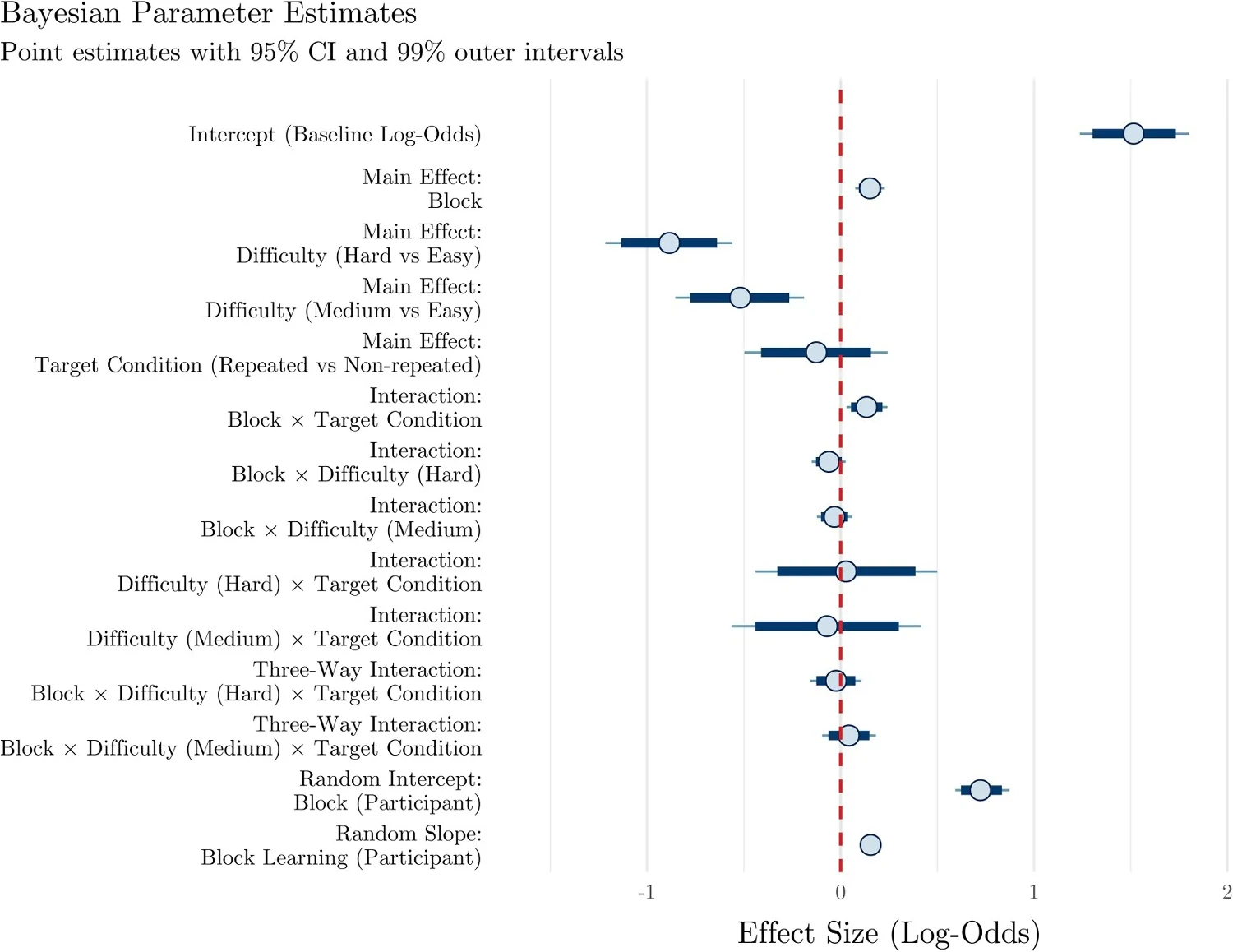
Posterior parameter estimates from hierarchical Bayesian logistic regression. Point estimates (*posterior medians*) with 95% (*thick lines*) and 99% (*thin lines*) credible intervals. All effects represent log-odds differences relative to baseline (easy difficulty, non-repeated targets, Block 1). The hierarchical structure accounts for participant-level variability in baseline performance (random intercept) and learning trajectories (random slope). Three-way interactions (*top*) quantify how repetition benefits depend on both block progression and difficulty. Uncertainty intervals derive from MCMC posterior draws across four chains

**Fig. 26:**
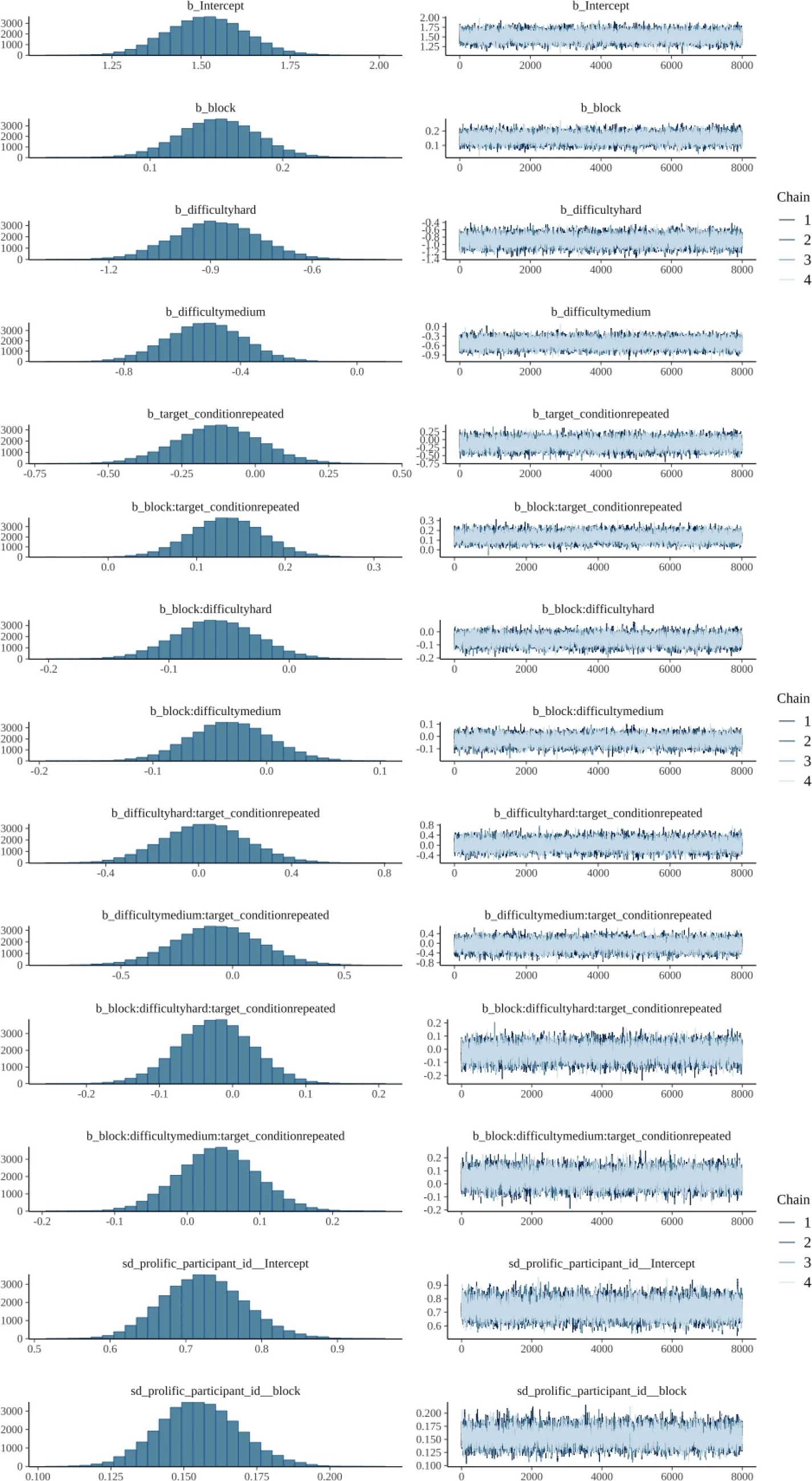
MCMC diagnostics plots

The mean accuracy for 240 participants across six blocks for repeated and non-repeated conditions at three difficulty levels (easy, medium, and hard) is shown in Fig. 6. Accuracy systematically varied with task difficulty, showing a clear graded pattern: performance was highest in the easy condition, intermediate in the medium condition, and lowest in the hard condition. Across all difficulty levels, mean accuracy for repeated targets started at the same level as non-repeated targets, and then outperformed them in later blocks. The graded accuracy distinctions were preserved throughout learning. We fitted a Bayesian hierarchical model to quantify these graded effects of task difficulty and stimulus repetition across blocks. All parameters and derived quantities showed robust convergence ($\hat{R}$ close to 1.00; bulk-ESS>8000; tail-ESS>15,000; see diagnostic plots in Fig. 26, Appendix B.3). Posterior parameter estimates are provided in Table 6 and visualized in Fig. 25 in Appendix B.3. A more detailed description of the modelling result is provided in Appendix B.3.

## Discussion

### Perceptual scaling and similarity judgement task

The interpolation procedure successfully generated sets of images with a gradient of perceptual similarity and without impacting the semantic meaning across image variations excessively. The use of text prompts and text-to-image model allowed for very high flexibility in the choice of categories. Keeping the text prompt constant while interpolating between the starting noise latents proved to be an effective and controlled approach for manipulating the perceptual characteristics of a scene while maintaining its semantic meaning.

Our procedure synthesized a set of high-resolution naturalistic object stimuli from different categories that are systematically ordered along a psychophysically validated perceptual continuum. They could prove useful for many cognitive tasks that involve measurements on perceptual continua, such as change detection or working memory tasks  (Zhang et al., 2008). In comparison to scraping images from the internet (Hebart et al., 2019), or using pre-trained GANs (Son et al., 2022), using a text-to-image model in combination with psychophysical ordering also showed extreme versatility in semantic contents and high image quality. This method could be used to generate new images and categories, tailoring them to specific experimental needs.

Previous image sequences and data sets have typically used considerably different exemplars (Greene & Oliva, 2009; Krizhevsky et al., 2012; Lin et al., 2014; Hebart et al., 2019). In contrast, our stimulus set is primarily intended for paradigms that aim for local confusability and threshold-level variations, as exemplified in our memory validation study. For each object-scene, we generated a set of image variations that (i) can be placed on a monotonic perceptual continuum, (ii) preserve the same semantics (e.g., “picture of a cat on a carpet”), and (iii) differ only in low-level perceptual details. Please note that our approach in its current form does not aim to control the specific dimensions along which an image is varied. For synthetically generated images as here this can be achieved by using either extensions of diffusion models (Gandikota et al., 2024) or GANs (Goodfellow et al., 2014). One possible future application could be the augmentation of existing datasets. For example, one could use the semantic categorization of the THINGS dataset (Hebart et al., 2019), which was implemented using a data-driven method, to generate a higher number of exemplars for each category. Another possible application is the extension of work on natural scene properties (Greene & Oliva, 2009), for example, by implementing the possibility of varying specific feature dimensions (Gandikota et al., 2024).

Despite their photorealism, our synthetic images do not fully resemble (randomly sampled) snapshots of real-world scenes. Our synthetic stimuli capture a narrower range of the variability that characterizes natural scenes. Our aim was to provide a visually consistent stimulus set, in terms of appearance and image layout across categories. However, such choices consequently produced “idealized” versions of the scenes and reduced variation in factors such as contextual complexity, lighting conditions, and overall naturalness. Importantly, our object-centered layout deliberately placed a single, prominent object at the center of the image. This design choice preserves clear semantics and limits contextual clutter, in line with object-centric datasets such as THINGS (Hebart et al., 2019) and neuroimaging work using ImageNet (Chang et al., 2019; Gong et al., 2023). While central placement exploits the well-documented oculomotor ‘center bias’ in scene viewing (Tatler, 2007), it also limits the stimulus set’s applicability for researchers interested in maximally realistic natural scene perception (see e.g., Greene & Oliva, 2009; Epstein & Baker, 2019; Engeser et al., 2025). Our stimulus set is in fact designed for research on object-based attention, perception and memory, rather than for complex, cluttered multi-object scenes (Greene & Oliva, 2009). Researchers interested in studying scene perception should weigh this trade-off when deciding whether the stimulus set is appropriate for their experimental needs. Future work could extend the pipeline to generate scene variants to study cognitive functions such as naturalistic gaze behavior for complex scenes.

The LPIPS metric (Zhang et al., 2018) played a central role as a quantifiable prediction of perceptual similarity and enabled us to efficiently manage and screen a large set of images before fine-tuning the ordering based on psychophysics. In general, LPIPS showed a good alignment with the similarity judgements from the online task and proved to be a valid first approximation. One limitation of our approach is the lack of a comparable baseline for object scene similarity. Although we found the score to be reliable and aligned with human perception within a single object scene (e.g., indicating a graded increase in perceptual dissimilarity), the metric magnitude across object scenes was less interpretable. Future work could address this limitation by accounting for similarity scores not only within but also across object scenes.

One limitation of the present pipeline is that both the high-level categories (natural vs. artificial) as well as the specific objects (e.g., “butterfly,” “sailboat”) were chosen *a priori* by the experimenters, leaving room for selection bias. To address the subjectivity inherent in prompt selection, future work could combine generative image synthesis with data-driven concept-selection frameworks to represent semantic spaces more uniformly. Another question is whether similar text prompts could also be used to identify and retrieve images from web search engines. Compared to such web-based retrieval of existing images, we believe the use of image synthesis offers several key advantages: First, it allows to flexibly generate arbitrary stimuli that might not be available on the Internet (Podell et al., 2023). Second, it provides a fully parametrized framework that allows images to be generated with a variable number and similarity, beyond what is available in image databases (Hebart et al., 2019; Krizhevsky et al., 2012; Lin et al., 2014).

As is common for AI-generated images (Cao et al., 2024), we also identified artefacts, which exhibited high variability across categories. Although the exclusion rates were generally low, some objects were more prone to the presence of artefacts than others. This variability can be attributed to several factors. A first possibility is that there were differences in the availability of training data for different objects (Yang et al., 2023). Another possibility is the challenge of finding effective prompts (Liu & Chilton, 2023), given the virtually infinite number of possibilities. Finally, the levels of familiarity vary across images, which may make artefacts of more familiar objects more noticeable. The category with the highest incidence of artefacts was *animals*, followed by *items* and *vehicles*. For example, *giraffe* images had the highest exclusion rate, often due to inaccuracies such as an incorrect number of legs, which is a very salient feature (Kamali et al., 2025). Also, there might be tighter normative constraints of features for certain objects, for example, the number of legs for a giraffe is fixed, whereas the number of leaves on a plant might be more variable. These are all known problems in generative AI, although models are quickly improving (Cao et al., 2024). In contrast, artefacts were less frequently identified in images of plants, landscape elements, and buildings (which are less normatively constrained). Although we did not investigate the cognitive implications of these category-specific differences empirically, such variability may be informative for future studies investigating models of human and AI cognition (Lu et al., 2023; Huang et al., 2024; Kamali et al., 2025). In particular, some categories might have very rigid norms for specific features (e.g., the fixed number of extremities in animal anatomy) and pose greater challenges for generative models than less constrained categories (e.g., trees, where the number of branches is not so constrained). Future work could examine the alignment between generative models and humans in how visual features are represented and integrated across semantic categories. From a practical perspective, category-dependent differences should be explicitly considered when selecting target images in line with experimental goals. Object scenes with smoother interpolations may be preferable for tasks focused on graded perceptual continua or memory precision, whereas object scenes with structurally constrained features may be more suited for studies investigating feature-binding processes or anomaly detection. Another limitation of our approach is that the exclusion criteria were rather subjective and future work could use crowd-sourcing platforms or artefact classifiers (Cao et al., 2024) to tackle the problem systematically.

### Memory validation task

We validated our stimulus set in an additional behavioral experiment, investigating how the precision of scene encoding in visual working memory (vWM) varies as a function of proximity along our modelled continua. We observed a clear gradient with more similar images being memorized better. Despite the fact that performance increased with repeated experience with the objects (see Fig. 6, bottom right), the similarity-related gradient was largely maintained. VWM performance is known to benefit from contributions of LTM, where aspects such as meaning, familiarity, and previous exposure strengthen memory representations (Xie & Zhang, 2018; Schurgin et al., 2020; Brady & Störmer, 2022). In paradigms rooted in Hebbian principles (Hebb, 1961), repeated exposure reinforces stimulus-specific traces in both vWM and LTM, enabling sequential recall improvements through synaptic reinforcement (Johnson & Miles, 2019; Mızrak & Oberauer, 2022; Souza & Oberauer, 2022). Continuous feature-report tasks show that, under repeated exposure, LTM can support high-fidelity representations with precision comparable to that of vWM (Brady et al., 2013; Miner et al., 2020). Note that performance improved across all difficulty levels, even if object scenes were not repeated. We interpret the overall pattern as the joint expression of (i) a stimulus-specific component (repetition-based strengthening of long-term memory traces for individual scenes) and (ii) a stimulus-nonspecific component (general adaptation to the task) (Sagi, 2011). Future studies could further minimize nonspecific effects by extending the practice phase until accuracy stabilizes across a broad range of object-scene pairs.

Our results align with previous findings linking latent space distances to working memory performance (Son et al., 2022; Bates et al.., 2024), but extend the investigation to a broader range of object categories. The systematic manipulation of perceptual similarity allowed us to directly assess how prior exposure strengthens LTM representations and facilitates recognition, particularly in high-interference conditions where targets and foils were most confusable. This level of experimental control was essential for isolating the contribution of LTM to WM, a distinction typically challenging to capture with naturalistic images due to their inherent variability and complexity.

### Conclusion

The use of generative models for stimulus synthesis has the potential to set a new standard in cognitive research by enabling the creation of customized, naturalistic stimuli and psychometrically ordered stimuli tailored to the needs of cognitive experiments. These models can be highly useful in addressing the trade-off between ecological validity and experimental control. Here, we introduced a novel approach using diffusion-based generative models, specifically Stable Diffusion XL, in combination with flexible text prompts to synthesize a stimulus set of high-resolution, photorealistic scenes with graded perceptual differences and consistent semantic content.

Our three-step validation procedure demonstrated that our stimuli effectively captured a perceptual continuum, first quantified objectively using the LPIPS metric (Zhang et al., 2018) and subsequently confirmed through human psychophysical judgements collected in an online task. Moreover, this perceptual scaling was further validated in a visual working memory task, where recognition accuracy decreased systematically as perceptual similarity between targets and foils increased. Performance was highest in the Easy condition and progressively lower in the Medium and Hard conditions, where foils more closely resembled the target. This pattern validates that proximity along the perceptual continua affects working memory performance in a graded way.

By providing the stimulus set publicly (see Data availability section), we hope to contribute a valuable tool for the research community, bridging the gap between ecological validity and experimental control in visual cognition studies. These stimuli are particularly well suited for addressing key challenges in visual cognitive research, including change detection, memory, and object recognition.

## Figures and Tables

**Fig. 7:**
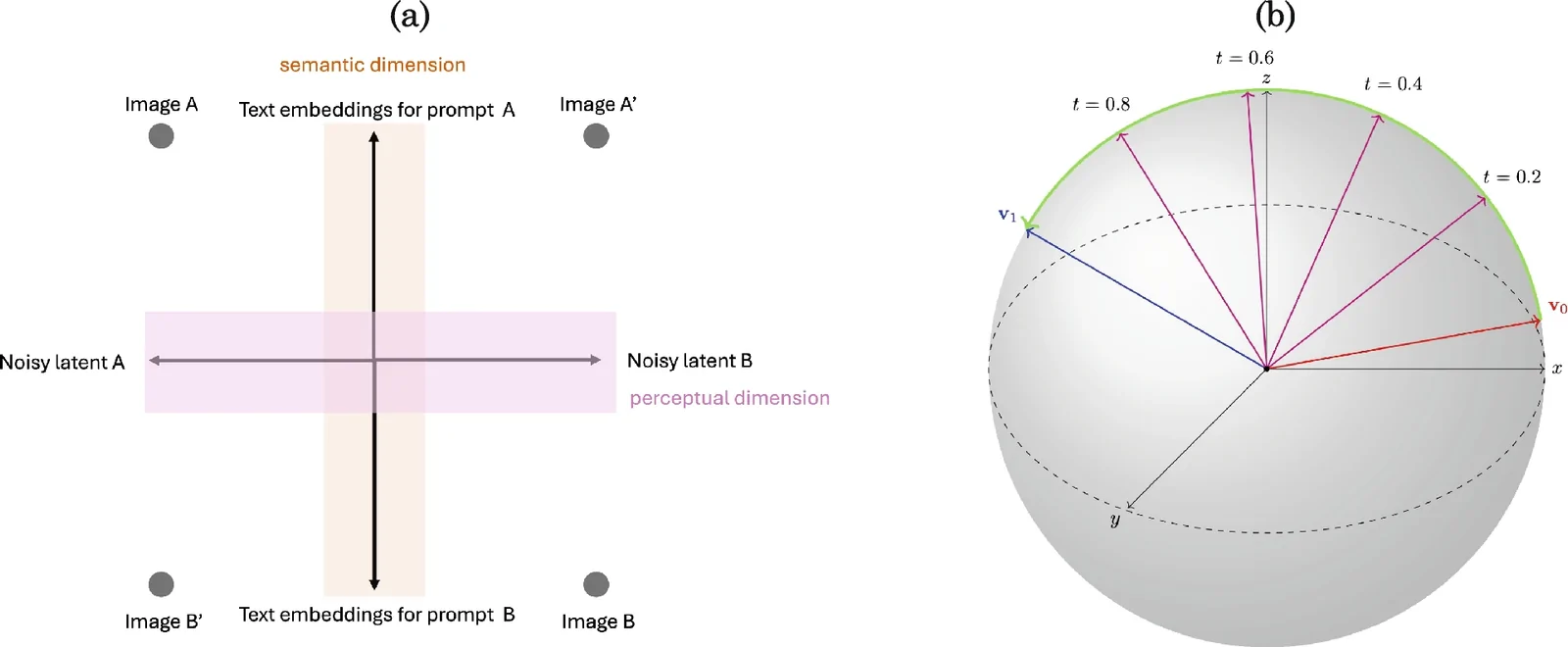
**a** Schematic representation of the two possible spaces in which the interpolation can be operated. It is possible to interpolate either between the text embeddings (encoded by the CLIP models) or the initial noisy latents. Interpolation between text embeddings will result in significant semantic variations (e.g., between Image A and Image B, or B’), whereas interpolations between noisy latents will mostly involve perceptual variations, keeping the meaning of the image fairly constant (e.g., between Image A and A’ or Image B and B’). It should be noted that this is a simplification because semantic variations cause perceptual variations and vice versa. The aim here is to highlight the main dimension of variation. Moreover, to have fine-grained control of the scene layout, methods relying on models like ControlNet should be used. **b**) A three-dimensional simplification of the spherical linear interpolation (slerp) between two vectors $v_0$ and $v_1$. In this study, the interpolation is applied between $128 \times 128 \times 4 \times 1$ tensors

**Fig. 9:**
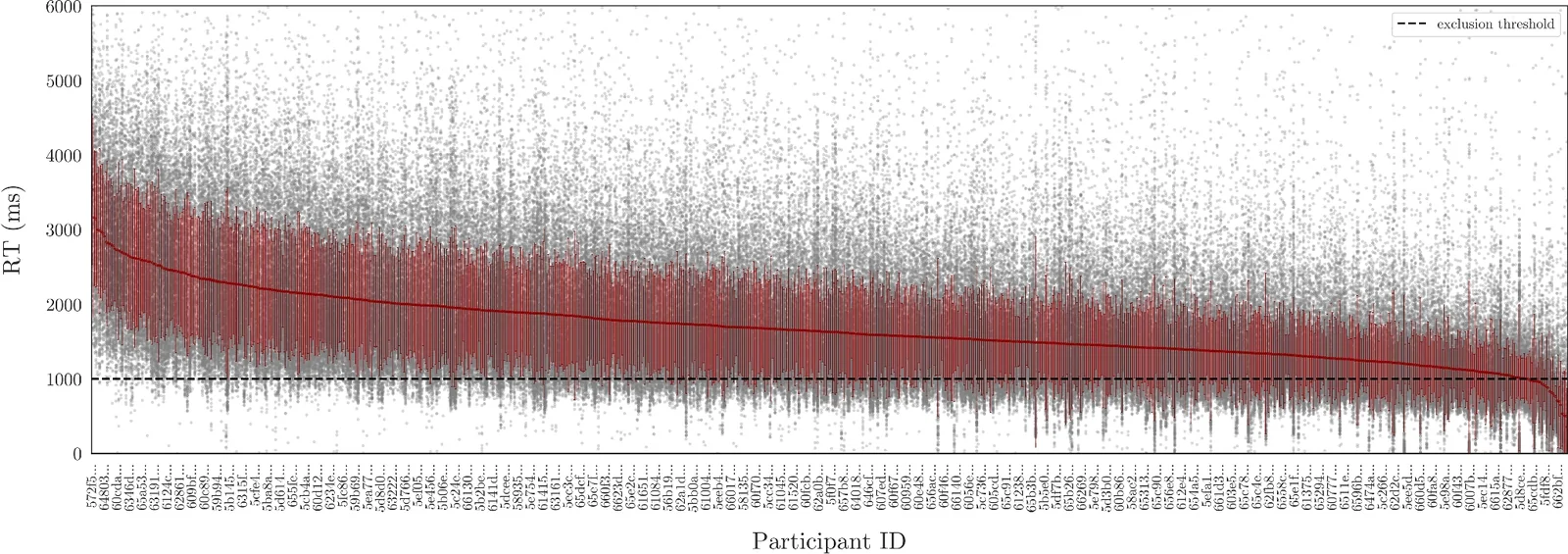
Distribution of reaction times for all participants, ordered by mean reaction time. The 1000-ms cutoff threshold is indicated by the *dashed line*

**Fig. 10:**
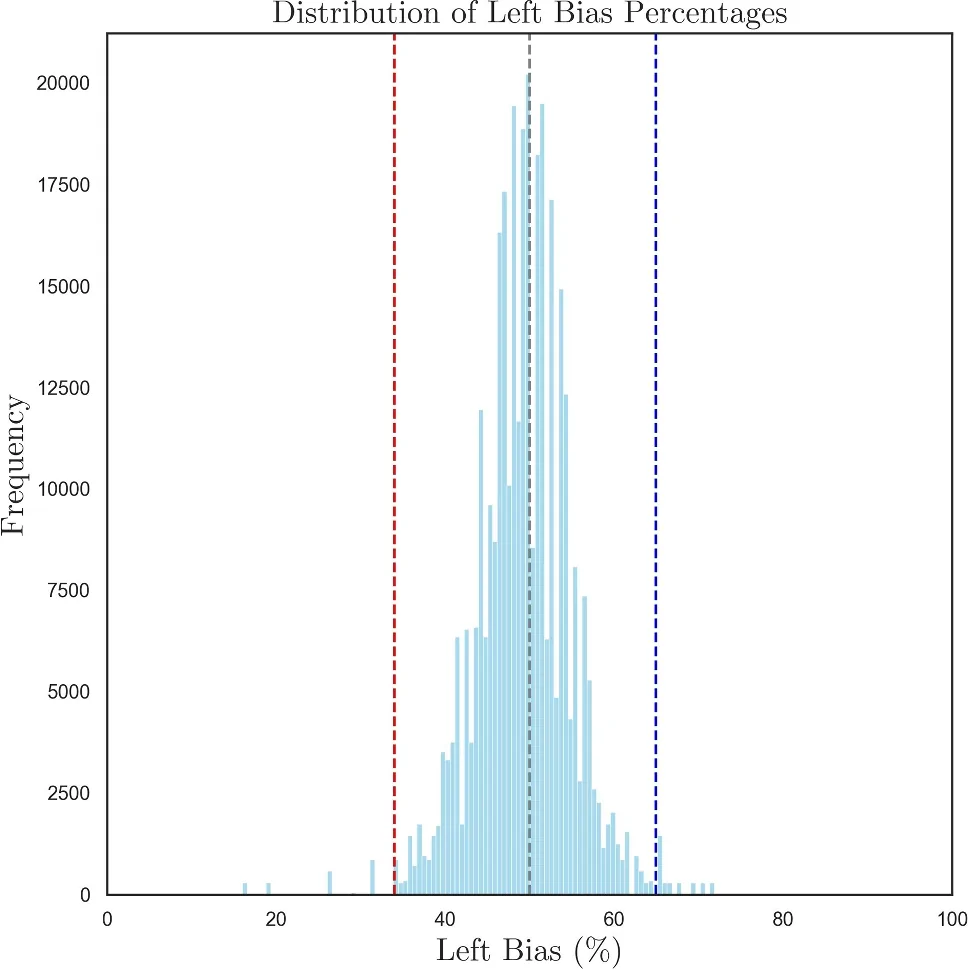
Distribution of left bias percentages across participants. The *dashed blue* and *red lines* indicate the thresholds for extreme biases, while the central *black dashed line* represents the expected 50% bias

**Fig. 11:**
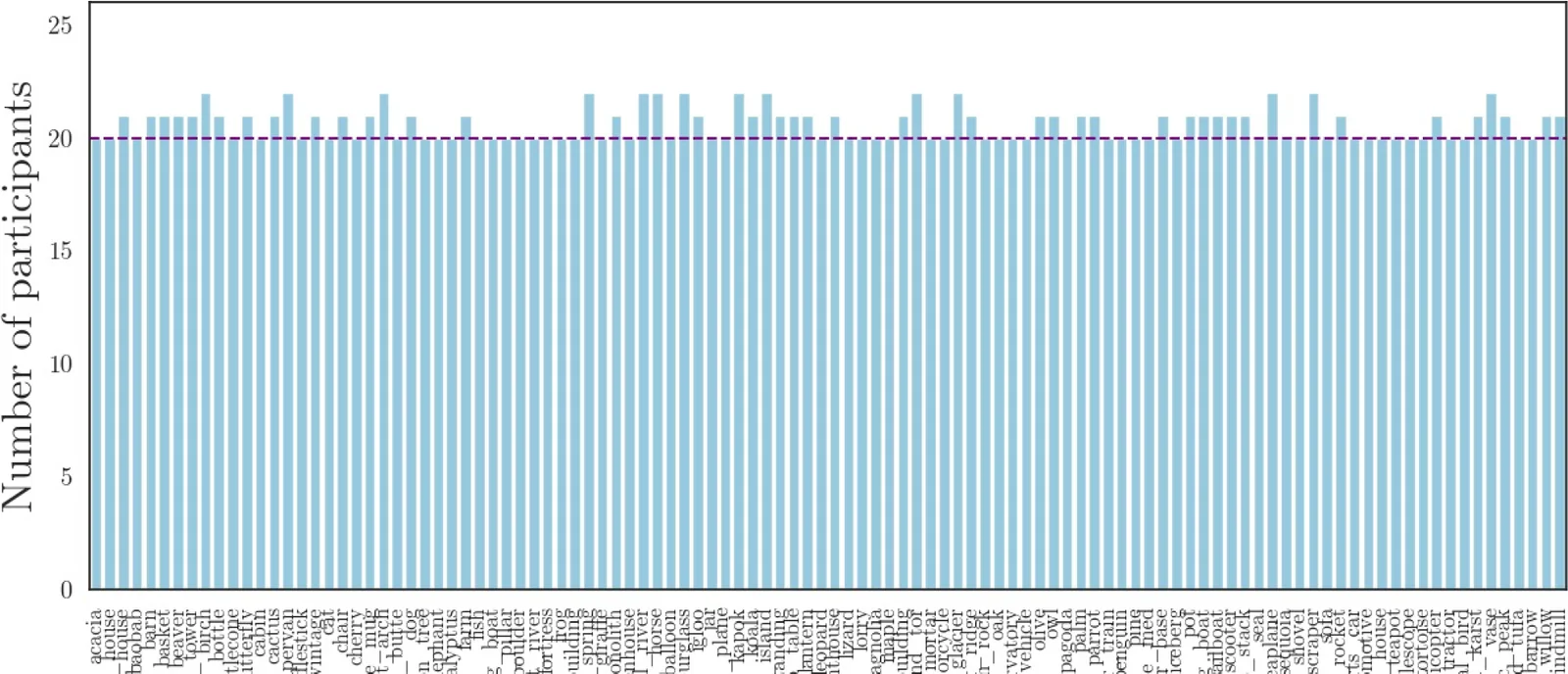
Participant count for each object

**Fig. 12:**
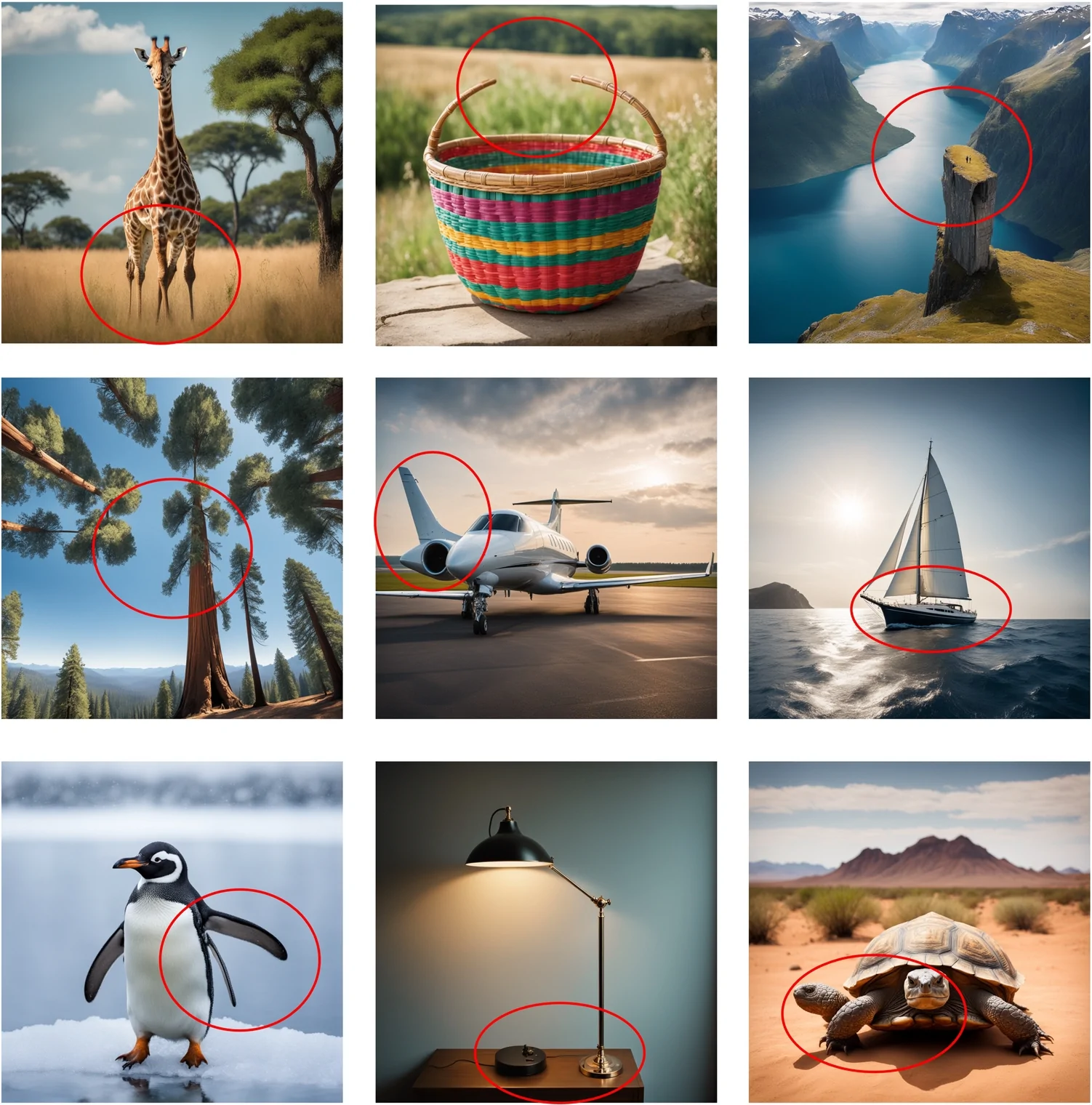
Some examples of excluded exemplars, highlighting the artefacts. A common artefact when generating animals was the wrong number of limbs (*giraffe* and *penguin*) or other body parts (the *head* of the *tortoise*). Other artefacts depicted here are layouts that did not follow the prompt (e.g., the *sequoia*), non-realistic perspectives (e.g., the horizon in *sailboat*), or compositions (e.g., the handle in *basket*, the reactor in *jet plane*, and the lamp stand in *lamp (table)*). Finally, despite the negative prompt, sometimes human figures made an appearance (e.g., in *fjord pillar*). Such images were also excluded

**Fig. 14:**
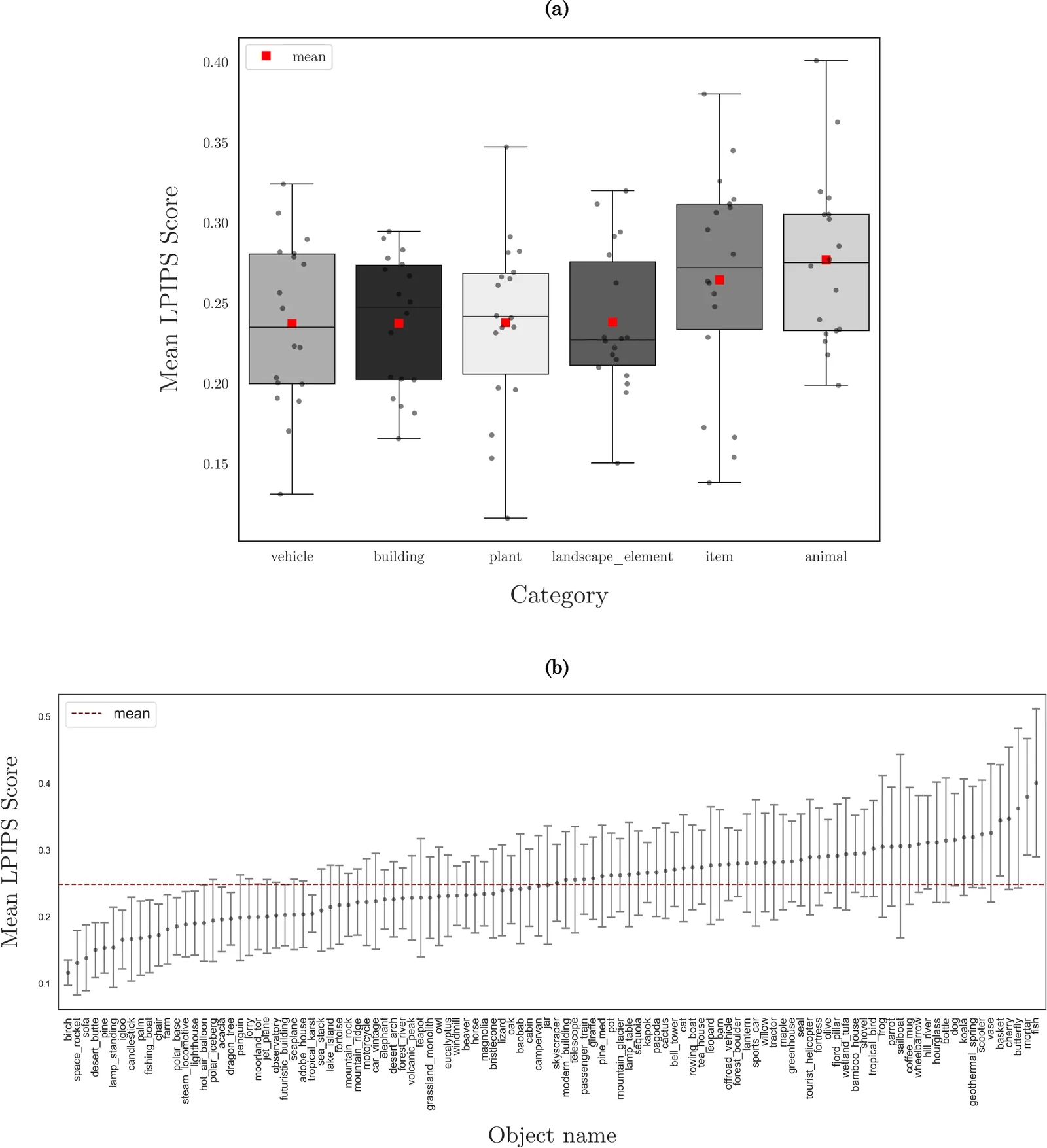
Mean LPIPS scores between exemplar images, grouped by **a** category and **b** object

**Fig. 15:**
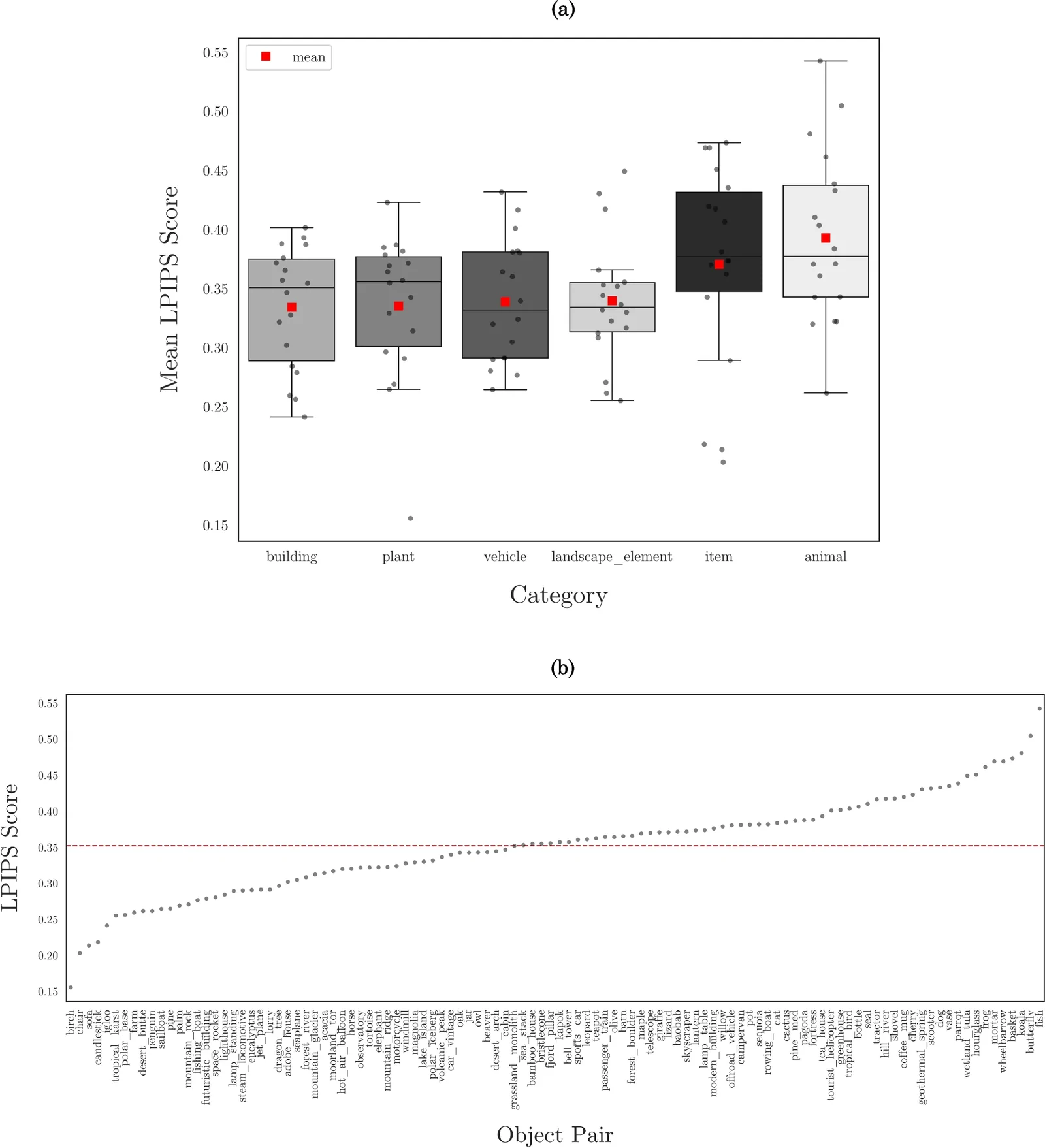
Mean LPIPS scores for selected pairs, grouped by object and category. **a** Distribution of mean LPIPS scores by category. Each data point is the mean LPIPS score for an object belonging to that category (18 data points in total). The category means are shown in *red *. **b** Mean LPIPS scores by object, ordered by magnitude. Lower scores indicate more homogeneity (i.e., highest similarity) between the exemplar images. The *red dotted line* is the overall mean across all similarity scores

**Fig. 17:**
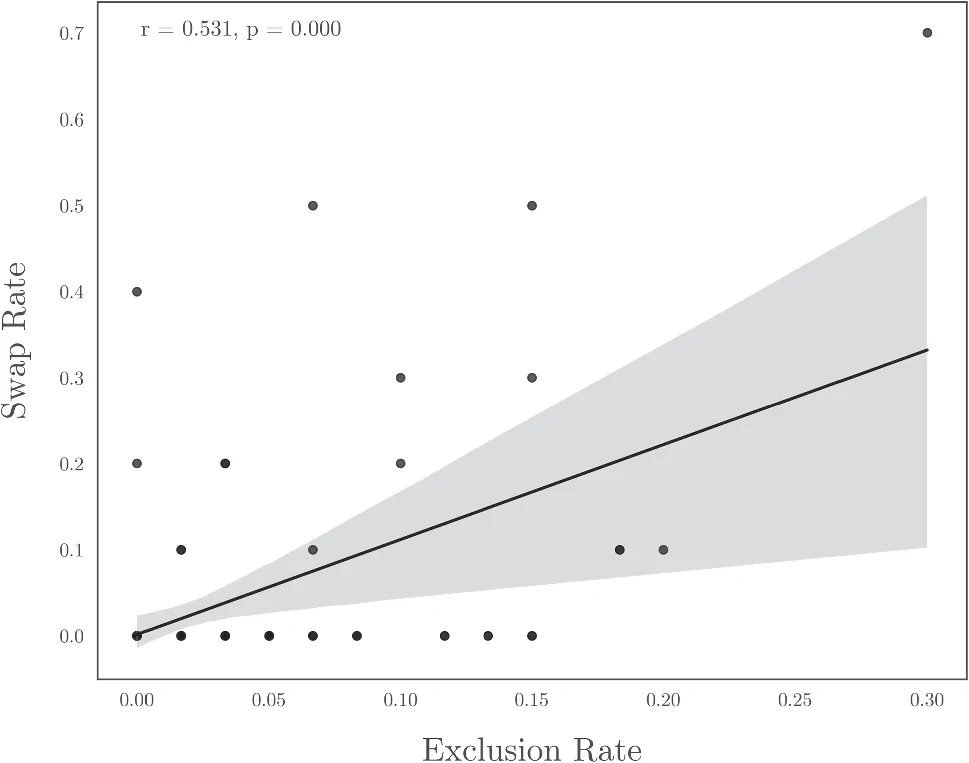
Correlation between exclusion and swap rates

**Fig. 18:**
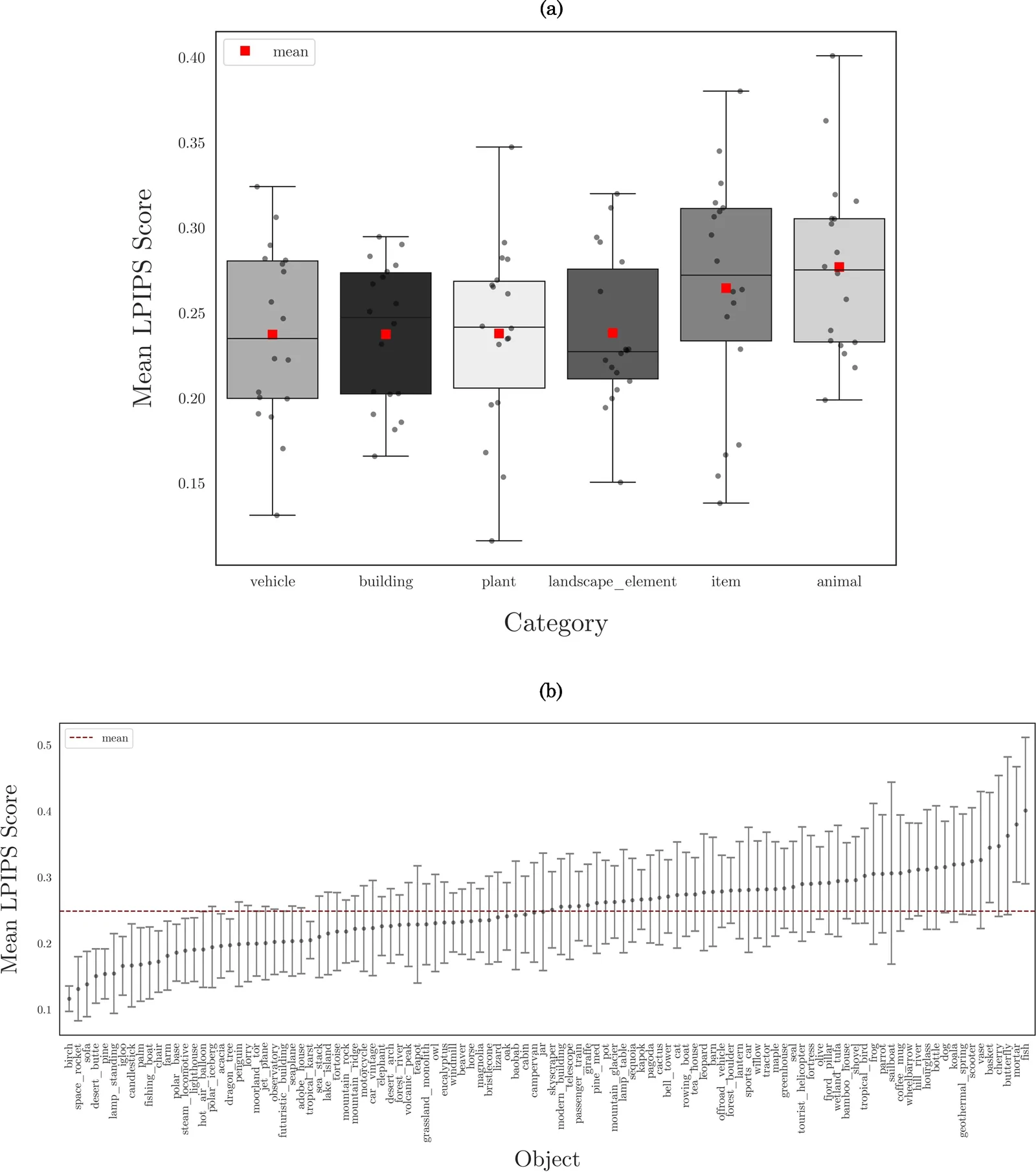
Mean LPIPS scores between *anchor images* and each interpolated image, grouped by **a** category and **b** single objects

**Fig. 19:**
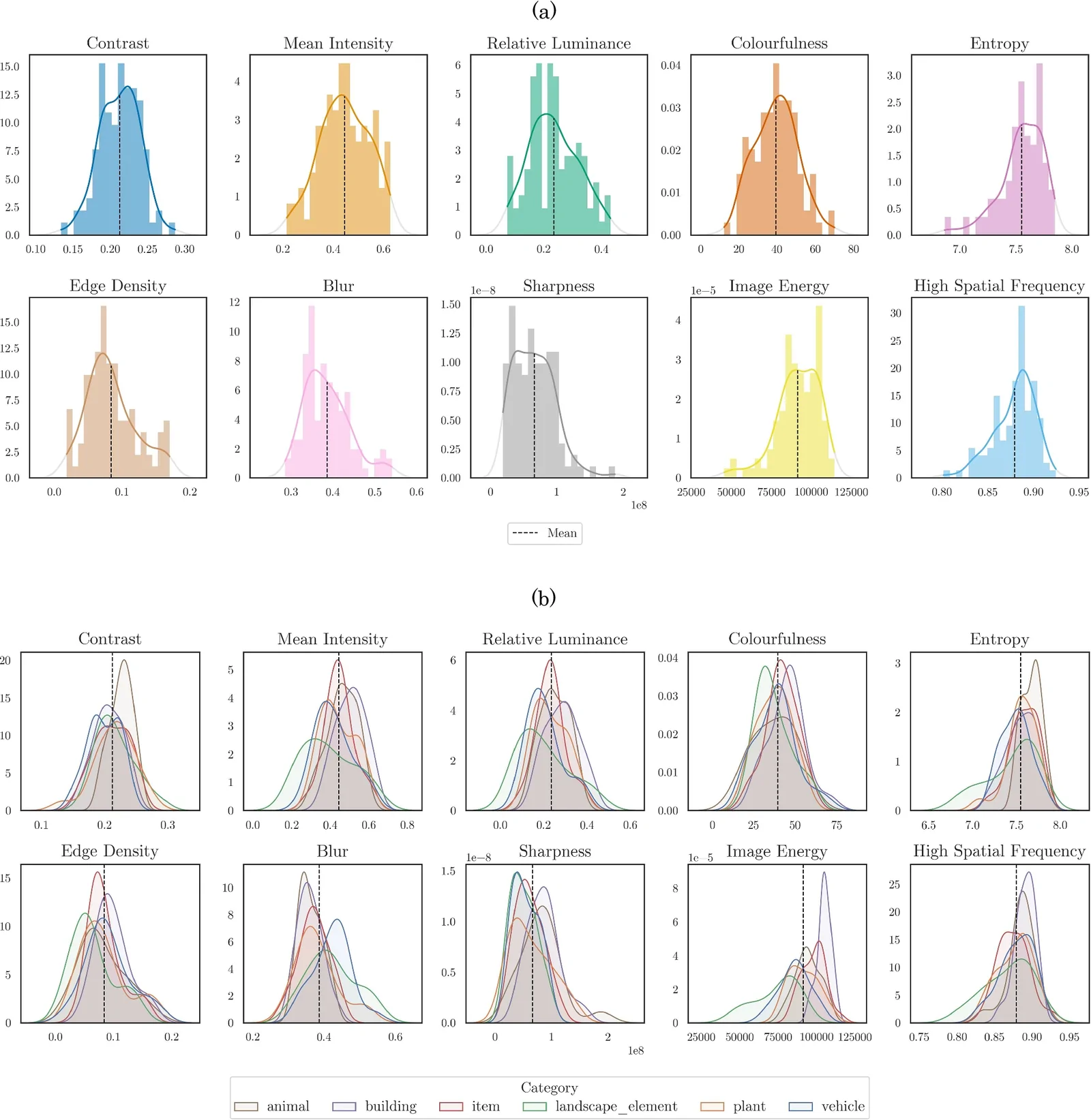
Physical characteristics of the *anchor images* of the final stimulus set. **a** The first set of histograms illustrates the overall distributions of various image features across the stimulus set. Each feature is accompanied by a fitted density curve and a dashed line indicating the mean value. **b** The second set of plots provides a comparison of feature distributions across different image categories (animal, building, item, landscape element, plant, vehicle). Each plot shows the distribution of a feature for each category, with the overall mean indicated by a *dahed line*

**Fig. 20:**
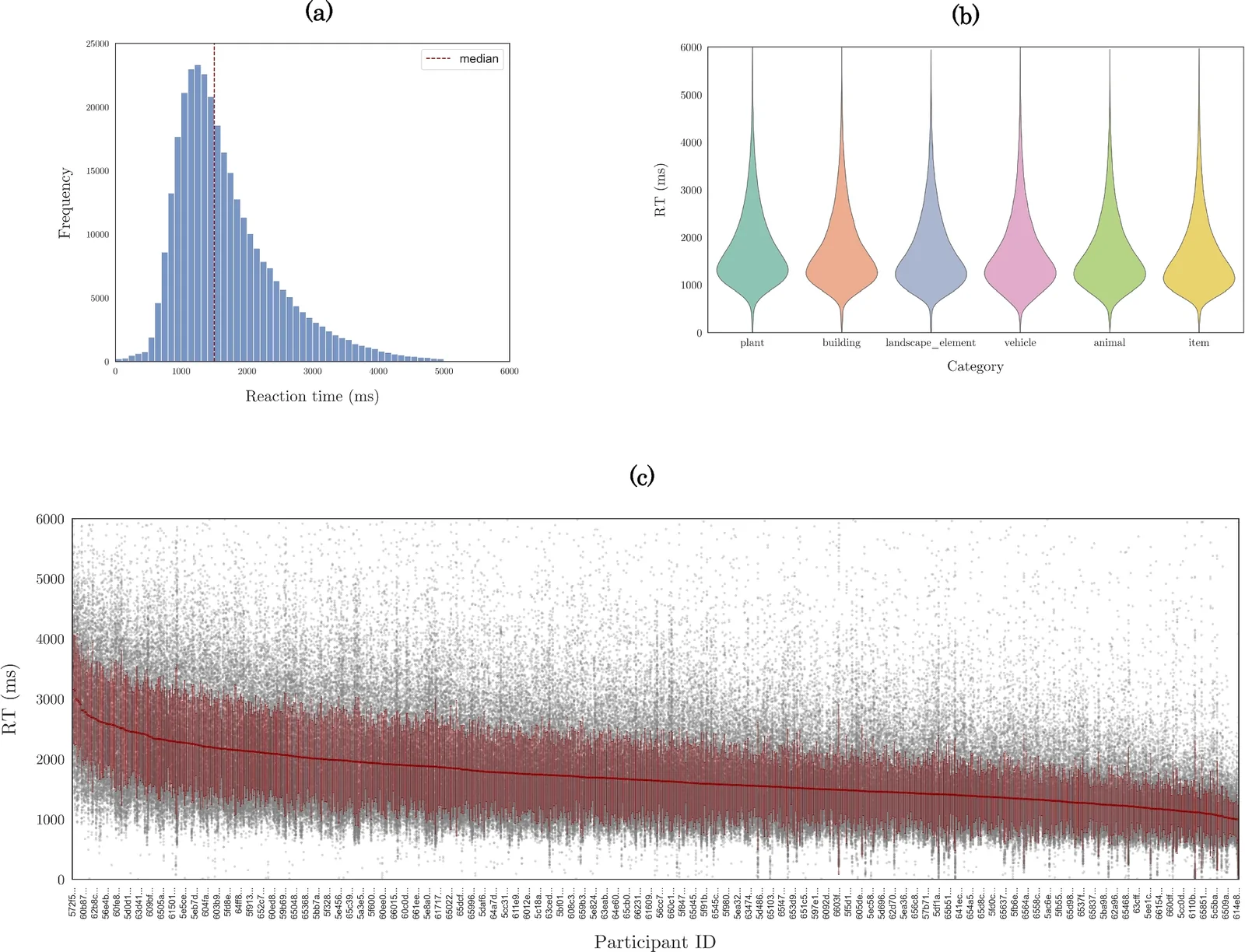
Distributions aggregated over all participants’ data: **a** Reaction time distribution, **b** reaction time distributions for each category, and **c** reaction time distributions for all participants in the final sample ($RT < 6000 \, \text {ms}$), sorted by participant ID in descending order of mean reaction time

**Fig. 21:**
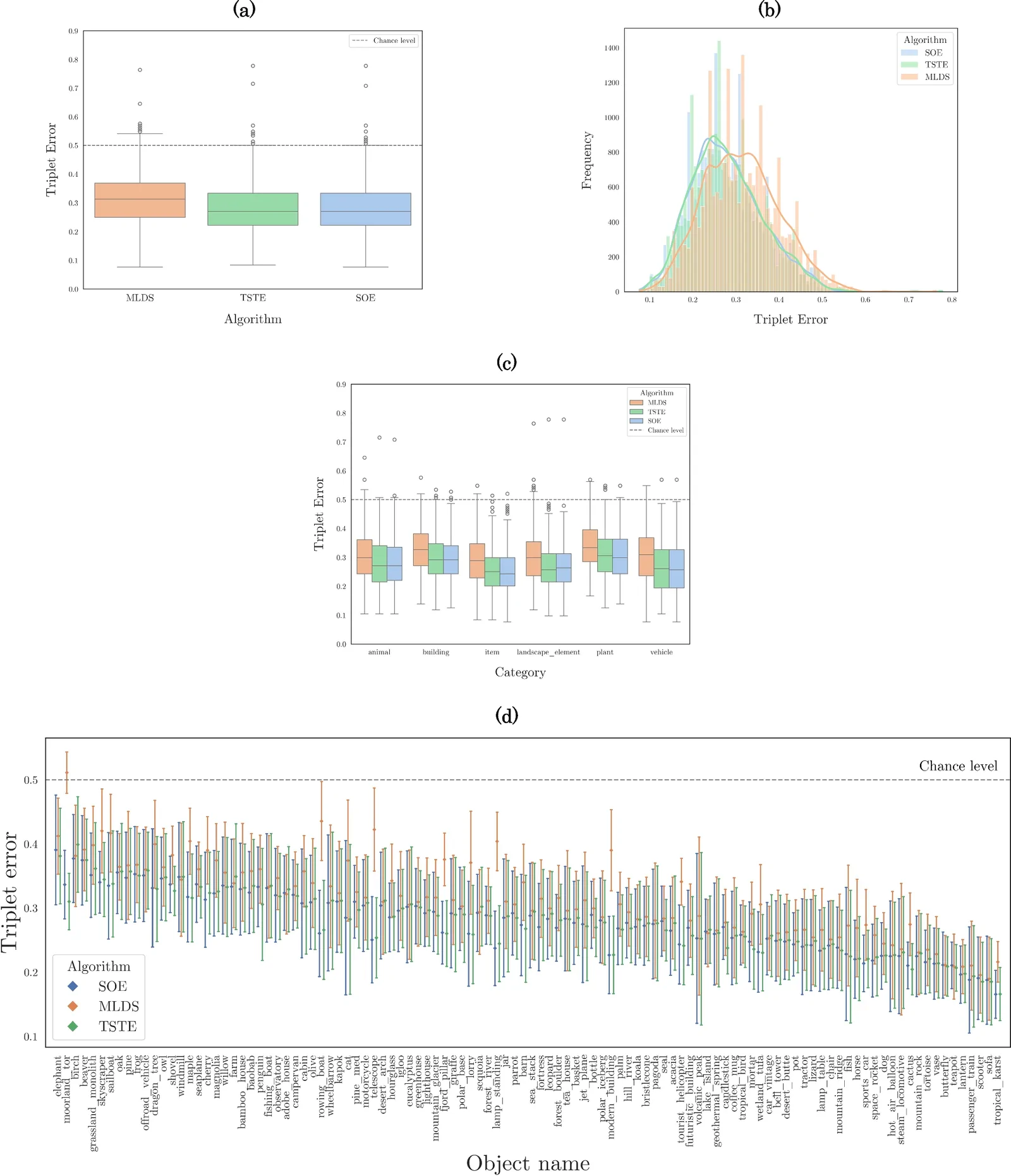
Comparative performance analysis of SOE, *t*-STE, and MLDS algorithms using triplet error. **a** Overall performance: mean triplet error across conditions. **b** Error distributions: variability per algorithm as histograms. **c** Category-specific performance: triplet error across object categories. **d** Object-specific performance: triplet error for individual objects

**Fig. 22:**
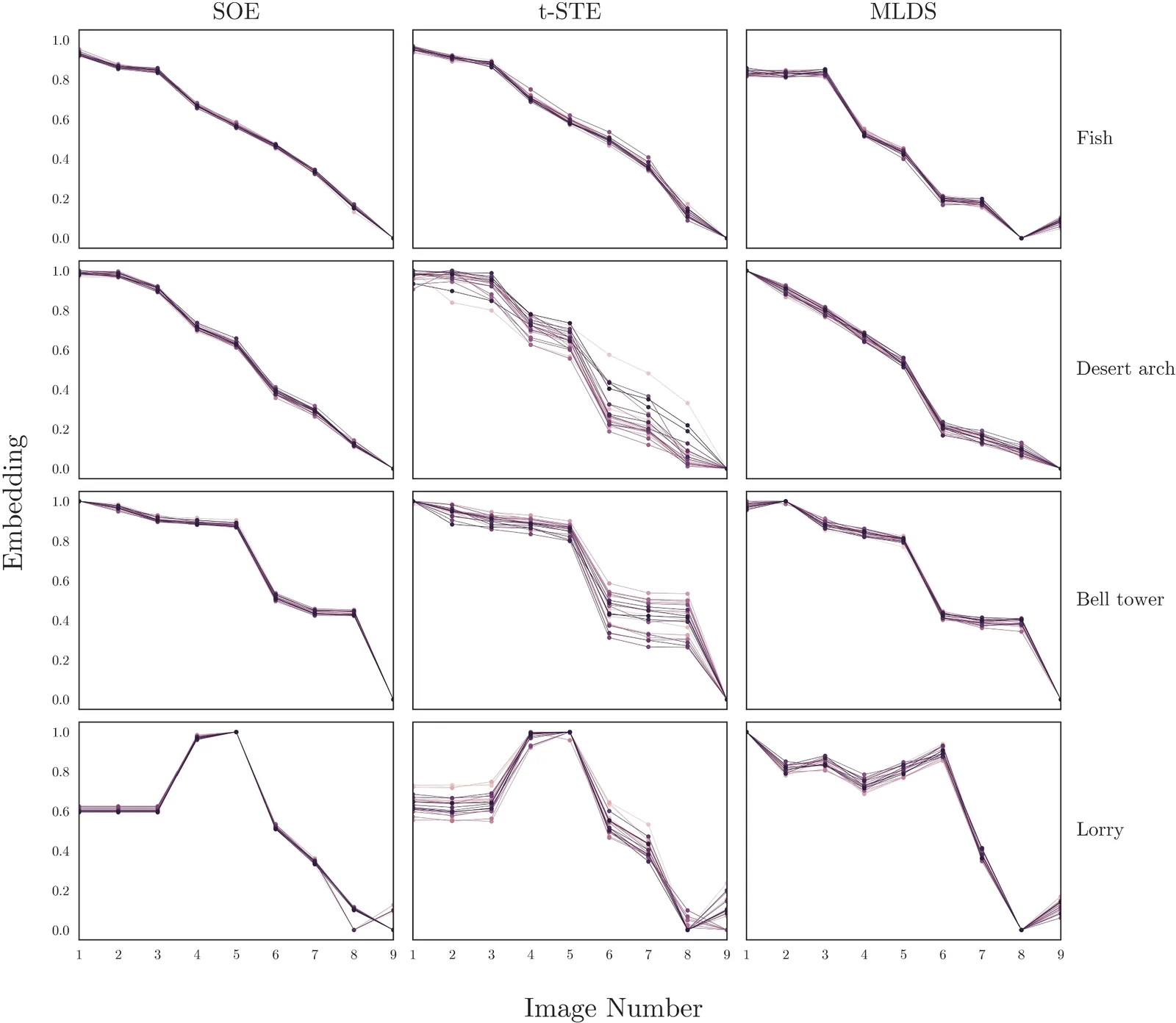
Visualization of the variability of embedding estimates across algorithms for **a**) *desert arch*, **b**) *fish*, **c**) *bell tower*, **d**) *lorry*. Each line represents embedding values estimated from one cross-validation fold, where one participant is left out. This illustrates the sensitivity of the estimates to individual participants. SOE yields more robust estimates compared to the other algorithms, though all perform well overall. The image order on the *x*-axis follows the model-based similarity metric (LPIPS), not the participants’ reordered judgements. This explains why the lines are not monotonic, as the model ranking does not necessarily correspond to the participants’ evaluations

**Fig. 23:**
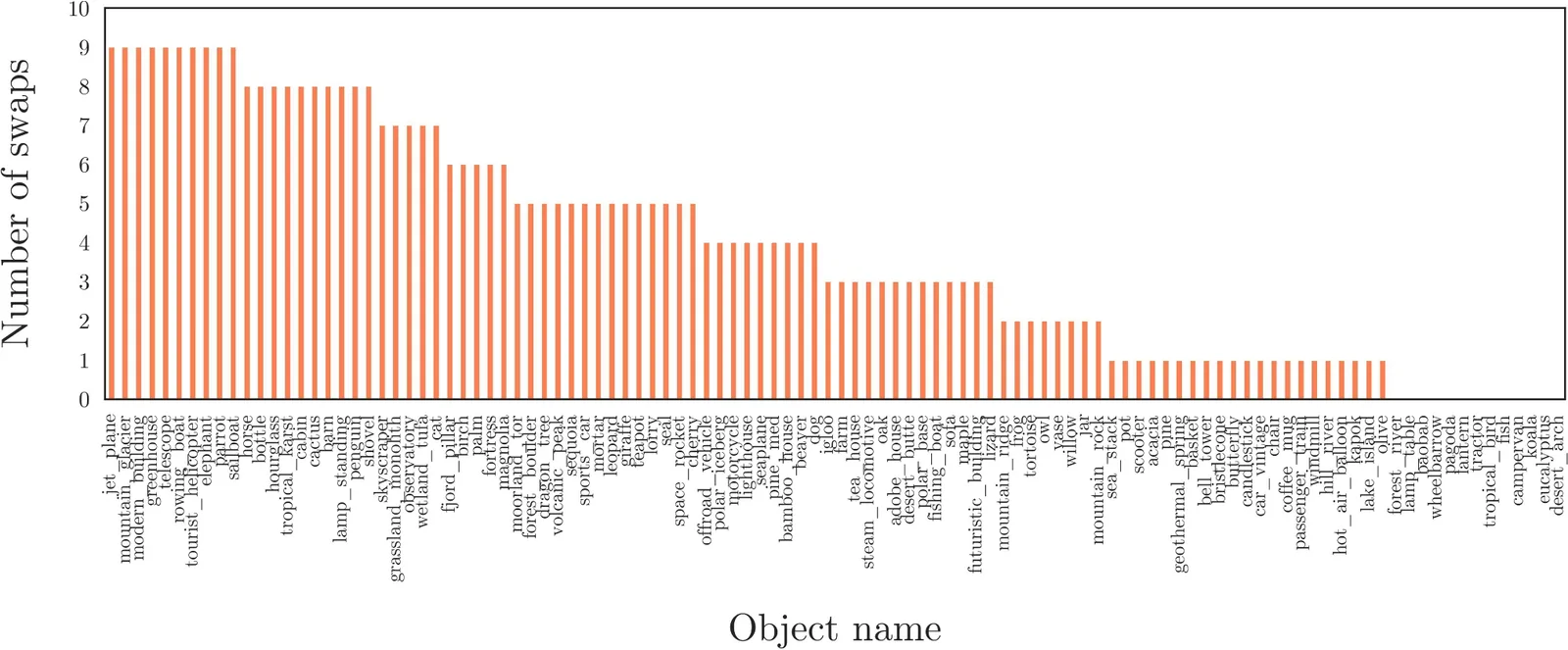
Reordering count by object

## Data Availability

The code, stimulus set (including images and metadata), and behavioral data from the online task will be made available on OSF at the following link: https://osf.io/pf4tv.

## Appendix A: Extended methods

### A.1 Image generation

In the next section, we provide detailed information about the model and its parameters. We will then describe the criteria used to generate exemplar images, followed by an explanation of how fine-grained image variations were obtained through interpolation between initial noise latents. A schematic overview of the procedure is shown in Fig. 1 (main text).

#### A.1.1 Model

Text-to-image diffusion models such as Stable Diffusion (Rombach et al., 2022) have gained considerable interest in the past years. They rely on two fundamental components to generate images: a starting noisy latent and a textual prompt (Yang et al., 2023). The noisy latent provides an initial random state, which the model progressively refines through a series of denoising steps, outputting an image. The textual prompt guides the model, conditioning the denoising process to generate images that align with the described content. Several text-to-image models are available on the market. We opted for Stable Diffusion XL (SDXL) (Podell et al., 2023) for two main reasons: output quality and customizability. In comparison to previous versions, SDXL relies on a larger UNet backbone and excels at generating high-quality images, especially at high resolutions (1024 x 1024 pixels). Moreover, the SDXL architecture incorporates two text encoders instead of one (OpenCLIP ViT-bigG and the original CLIP ViT-L text encoder), creating a richer textual conditioning for the diffusion process. Stable Diffusion XL benefits from a two-step generation process involving a base model and a refiner model, known as the ensemble of expert denoisers (Balaji et al., 2023): the base model generates a latent image, which is then fed into the refiner model. The refiner model completes the denoising process, enhancing the details of the image. In SDXL, the refiner model can be used to improve output quality of the fully refined image. The base and refiner model pipelines were instantiated from pretrained model weights, which were downloaded from the Hugging Face repository^1^. Images were generated using 50 inference steps for the base model to fully denoise the image and an additional 15 steps for the refiner model to improve detail quality. The default invisible watermark was added to each image to ensure it is marked as AI-generated. The ensemble of expert denoisers was integrated using a pre-trained low-rank adaptation (LoRA) (Hu et al., 2021) model^2^ to further improve the quality and detail of the generated images. Unlike the refiner model, which improves detail quality through additional inference steps, LoRA modifies the base model weights by adding a low-rank update matrix, allowing for efficient fine-tuning.

We used the Hugging Face diffusers 0.26.02 Python library, alongside standard libraries such as PyTorch 2.0.1 (Ansel et al., 2024), which provides high usability and customizability. We ran the model on the GPU cluster at the Bernstein Center for Computational Neuroscience, Charité-Universitätsmedizin Berlin, Germany. The cluster included four NVIDIA GeForce RTX 3090 GPUs (24GB each). To ensure efficient utilization of the GPU resources, we distributed the image generation tasks across the available GPUs using Pytorch Multiprocessing. Each GPU processed a single image independently.

#### A.1.2 Generation pipeline: design and diffusion of exemplar images

Generating images from textual prompts has the unprecedented advantage of letting users create any possible composition using natural language, but it also presents the problem of how to narrow down the prompt space to generate a finite and usable number of stimuli. We established the following criteria to generate our stimuli: first, they should have a clear subject, situated in the center and foreground (e.g., a picture of a cat sitting on the carpet); second, they should be situated in a coherent and realistic scene (e.g., an animal in its natural habitat); finally, there should be no human figures in the scenes.

Moreover, we divided the main subjects into six broad categories, three natural (“animals”, “plants”, “landscape elements”) and three artificial (“items”, “buildings”, “vehicles”). For each category, we defined a set of 18 objects (e.g., “cat”, “olive tree”, “sailboat”, “sofa” etc.), which we chose with the aim of creating a relatively diverse sample (e.g., animals from diverse biospheres, different kinds of landscape, etc.). All categories and corresponding objects are listed in Table 1. For each object, we generated 60 exemplars using the same prompt, but varying the starting noise tensor.

The structure of textual prompts was designed to balance two main aspects. On the one hand, the resulting images should vary across categories and be rich in detail. On the other hand, the whole stimulus set should have some consistency in terms of image layout. Therefore, we used relatively specific prompts, but with a shared main structure across the sample. With few exceptions, prompts started with a declaring the main subject (e.g., “film solitary bristlecone pine” or “photo of a barn owl” ), followed by information about its position (e.g., “in the foreground”, “in the center”) and about the background or scene it is situated in (e.g., “rocky terrain and blue sky in the background”), to finally describe the image style (“high-resolution photography”) and some additional attributes (e.g., “cinematic”, “bright light”, “beautiful”). In order to avoid the undesired addition of human figures, we also used a negative prompt (“people, person, human figures, human body parts, humans”), which was constant across all generations. We checked that this worked as intended by visual inspection. Using this procedure, for each object we generated a set of 60 various but semantically homogenous exemplars, maintaining its layout and general composition fairly consistent.

We initialized the starting noise latents of each exemplar using unique seed values (for 60 exemplars and 108 objects, 6480 seeds in total). Seeds were used to create a PyTorch Gaussian noise tensor with dimensions (1, 4, 128, 128), where 128 is determined by dividing the target resolution (1024) by the variational autoencoder (VAE) scale factor of eight used in Stable Diffusion XL. Storing the seeds ensured reproducible initialization of the noise latents, allowing the same image to be generated at later stages of the pipeline and interpolating between noise latents.

After generation, we visually inspected all images and excluded those with prominent artefacts (such as an animal with too many legs), especially in central positions. The reason is that they can be distracting, making the stimulus less suitable for a cognitive scientific experiment. However, we can provide these images upon reasonable request to researchers who may be interested in using them for further studies, such as those focusing on human perception of AI-generated artefacts.

**Table 1:** Overview of the six categories and their corresponding objects: three natural (animals, plants, landscape elements) and three artificial (items, buildings, vehicles), each with 18 diverse objects

Category	Objects
animal	beaver, butterfly, cat, dog, elephant, fish, frog, giraffe, horse, koala, leopard, lizard, owl, parrot, penguin, seal, tortoise, tropical_bird
building	adobe_house, bamboo_house, barn, bell_tower, cabin, farm, fortress, futuristic_building, greenhouse, igloo, lighthouse, modern_building, observatory, pagoda, polar_base, skyscraper, tea_house, windmill
item	basket, bottle, candlestick, chair, coffee_mug, hourglass, jar, lamp_standing, lamp_table, lantern, mortar, pot, shovel, sofa, teapot, telescope, vase, wheelbarrow
landscape_element	desert_arch, desert_butte, fjord_pillar, forest_boulder, forest_river, geothermal_spring, grassland_monolith, hill_river, lake_island, moorland_tor, mountain_glacier, mountain_ridge, mountain_rock, polar_iceberg, sea_stack, tropical_karst, volcanic_peak, wetland_tufa
plant	acacia, baobab, birch, bristlecone, cactus, cherry, dragon_tree, eucalyptus, kapok, magnolia, maple, oak, olive, palm, pine, pine_med, sequoia, willow
vehicle	campervan, car_vintage, fishing_boat, hot_air_balloon, jet_plane, lorry, motorcycle, offroad_vehicle, passenger_train, rowing_boat, sailboat, scooter, seaplane, space_rocket, sports_car, steam_locomotive, tourist_helicopter, tractor

#### A.1.3 Selection of the anchor images

In order to create variations of increasing dissimilarity from the exemplar image, we set up a procedure^3^ relying on the interpolation of the noise latents corresponding to a selected image pair. Each image pair included two anchor images, an *anchor image* and a *guide image* for the interpolation. These images were selected according to the following criteria:

1. The chosen images should be representative of the generated batch (e.g., no outliers).
2. They should be relatively similar to one another in terms of composition and perceptual features, avoiding changes in orientation or scene layout as much as possible.

Such criteria sought to optimize the interpolation smoothness (in the next step) while minimizing our manual interference with the process. In order to satisfy both conditions, for each object, we identified two images based on their relative perceptual similarity within the generated set. To quantify their perceptual similarity, we used the learned perceptual image patch similarity (LPIPS) metric (Zhang et al., 2018). The LPIPS metric quantifies the perceptual differences between images by comparing deep features extracted from a pre-trained neural network, and it has been shown to correlate well with human judgement (Zhang et al., 2018). For a few test images, we compared metrics using features from the SqueezeNet (Iandola et al., 2016), AlexNet (Krizhevsky et al., 2012), and VGG (Simonyan & Zisserman, 2015) architectures. The results across these models were comparable. Therefore, we opted to use SqueezeNet due to its computational efficiency and speed. We first computed the pairwise similarity of each image within the set. Then, for each image, we computed its mean LPIPS score against all other images in the set to assess its representativeness. The assumption behind this approach is that an image with a lower mean LPIPS score is, on average, more perceptually similar to the other images in the set. This higher similarity makes it a better representative of the overall set’s perceptual characteristics. The first image of the pair (the *anchor image*) was the image with the highest overall similarity to the rest of the set, that is, with the lowest mean LPIPS score. To select the *guide image*, we used the following procedure: first, we ranked images based on their mean LPIPS scores, and considered the images with the five lowest scores (excluding the score of the *anchor image*), which amounted to the top 8–10% of the image set, depending on how many images had been excluded. We then ranked the images within this subset by their similarity with the *anchor image*, selected the most similar as the *guide image* and the other ones as backup images. We then visually inspected the selected pairs. If the *guide image* had a significant visual disparity from the *anchor image* (e.g., opposite orientation), we selected the next most similar image as a backup. This process continued, if necessary, to the third most similar image and so forth, to ensure the pair maintained a high degree of perceptual coherence while maintaining the selection as objective as possible. In this way, we made sure that the interpolation would be computed between two images that are both representative of the set and similar to each other.

#### A.1.4 Interpolation algorithm

Once the two anchor images were selected, we generated the interpolations between the *anchor* and the *guide image* using their starting noise latents. In contrast to GANs, where the interpolation between two latent vectors corresponds to smooth image transitions, direct interpolation in diffusion is less straightforward. The interpolation between images can be operated either in the prompt space (i.e., between text embeddings), between the starting noise latents, or both (Fig. 7). Since the goal of this work was to maintain the semantic aspects of the images fairly constant, while changing their perceptual features, we interpolated between noise latents while maintaining the text embeddings constant^4^. Spherical linear interpolation (slerp) is particularly suitable for interpolating high-dimensional data as it preserves the length of the vectors and ensures smooth transitions (Shoemake, 1985). Given two vectors $\textbf{v}_0$ and $\textbf{v}_1$ and an interpolation parameter t where $0 \le t \le 1$, slerp can be defined as:

$$ \text {Slerp}(\textbf{v}_0, \textbf{v}_1; t) = \frac{\sin [(1 - t) \theta ]}{\sin [\theta ]} \textbf{v}_0 + \frac{\sin [t \theta ]}{\sin \theta } \textbf{v}_1 $$

where θ is the angle between $\textbf{v}_0$ and $\textbf{v}_1$, determined using the arccosine function on the normalized dot product between the vectors:

$$ \theta = \arccos \left( \frac{\textbf{v}_0 \cdot \textbf{v}_1}{\Vert \textbf{v}_0\Vert \Vert \textbf{v}_1\Vert }\right) $$

We first defined 200 interpolation values t or “steps”, which are linearly spaced between 0 and 1. The number of interpolation steps determines the granularity of this transition. We chose 200 steps as a compromise to balance between computational time and the smoothness of transition. Feeding the interpolation steps in the slerp function, we generated a series of intermediate noise latents that transition between the noise latents that were used to synthesize the *anchor image* and the *guide image*. We then used SDXL to denoise each interpolated latent and generate intermediate images. Importantly, we diffused the images iteratively in two sequential batches. In the first batch, we used the PyTorch generator initialized with the seed from the *anchor image* and diffused each intermediate noise tensor up to the midpoint. In the second batch, we used the PyTorch generator initialized with the seed from the *guide image* and diffused each intermediate noise tensor back to the midpoint. This dual-batch approach helped mitigate small variations or inconsistencies in the final output images, making sure that the diffusion process was equally controlled by both seeds.

#### A.1.5 Selection of the interpolated images

For each object, we selected ten variations, the *anchor image* and nine of its interpolations. To do so, we again used the LPIPS metric to compute the perceptual similarity between the *anchor image* and every interpolated image. We defined target similarity scores, linearly spaced between the minimum and maximum LPIPS values observed^5^. For each target score, we selected the image whose similarity score was closest to the target score, ensuring the absence of duplicates. This involves calculating the absolute differences between the similarity scores and the target scores, and selecting the closest matches. One option could have been defining the variations in terms of interpolation steps (e.g., generating ten interpolation steps in the previous step). Our approach, however, presents two advantages. First, the interpolation process in high-dimensional latent spaces can result in complex, non-linear changes in perceptual similarity. Consequently, simply selecting images based on fixed intervals of interpolation steps might not accurately represent a linear increase in dissimilarity from the *anchor image*. Our approach thus partially compensates for the non-linear perceptual changes by focusing on the actual observed similarities, rather than the potentially misleading interpolation steps. Secondly, the image generation process is susceptible to the introduction of artefacts. Since we wanted to make sure that the final image set maintained a high quality and, at the same time, a perceptual gradient, we conducted a final visual inspection to identify and exclude images with noticeable artefacts. If artefacts were detected, we selected immediate neighbors with very similar LPIPS scores as replacements. Therefore, generating a large number of interpolated images allowed us to exclude interpolated images with significant artefacts in favor of neighboring images, without affecting the perceptual gradient significantly and minimizing the interference of our own subjective judgement.

### A.2 Perceptual similarity judgement task

We used the LPIPS metric to pre-select ten images per object because it aligns reasonably well with human perceptual judgement (Zhang et al., 2018), and having participants evaluate all 108 objects with 200 images each would have been experimentally challenging. Although LPIPS provides a good approximation, we conducted a crowdsourcing task to experimentally validate the pre-selected images and adjust their ordinal positioning if needed.

#### A.2.1 Stimuli

The final stimulus set included a total of 1080 images, comprising 18 objects from six categories, with ten variations per object (see above for the selection procedure). Images were downscaled to 512 x 512 to decrease potential latencies from the client side, while maintaining high quality.

#### A.2.2 Similarity judgement task using triplet comparisons

Participants performed a similarity judgement task based on triplet comparisons, or “method of triads” (Torgerson, 1952), which is used both in psychophysics and machine learning (Aguilar et al., 2017; Demiralp et al., 2014; Haghiri et al., 2020; Künstle et al., 2022; Li et al., 2016; Wichmann et al., 2017). Given a set of stimuli $S = \{s_1, s_2, \ldots , s_n\}$, where n is the total number of stimuli, participants are asked to judge the similarity of three stimuli at a time. In particular, given a triplet of stimuli $(s_i, s_j, s_k)$, they are asked which between two probe stimuli $s_j$ and $s_k$ is most similar to a reference stimulus $s_i$. From the set of stimuli S, the number of all possible unique triplets is determined by the binomial coefficient:

$$ \left( {\begin{array}{c}n\\ 3\end{array}}\right) = \frac{n!}{3!(n-3)!} $$

However, since each element of the triplet can be a reference stimulus, the total number of possible triplet questions is obtained by multiplying the binomial coefficient by three:

$$ C = 3 \cdot \left( {\begin{array}{c}n\\ 3\end{array}}\right) $$

Since we planned to let participants assess the similarity between each combination of variations of the same object, but not across objects, in our case n was 10 (total number of image variations for each object) and the total number of triplet combinations C was 360.

Prior to data collection, we ran a set of simulations to estimate a) the number of triplet judgements that we would need to estimate object-specific psychophysical similarity functions robustly, and b) a target sample size of participants to judge each object. Previous work has shown that for n objects and d perceptual embedding dimensions, a subset of 2dnlog(n) triplets should be sufficient to reconstruct the embeddings with small error (Haghiri et al., 2020; Jamieson & Nowak, 2011; Jain et al., 2016), which for n=10 and d=1 amounts to $2 \cdot 1 \cdot 10 \log (10) = 20 \log (10) \approx 46$ triplets. Following the simulation results and expecting a relatively high noise for naturalistic images that are perceptually very similar, we estimated that a subset of 144 triplet judgements for one object from every single participant (40% of the total possible triplet combinations C) and at least 20 participants for each object would be necessary. The subset of triplet combinations was randomly sampled for each participant independently. Because of experimental constraints, i.e., not making the online task too long, each participant was assigned two objects. The assignment of the objects to participants was randomized.

#### A.2.3 Task procedure

After providing informed consent, participants received detailed instructions on the task procedure and were required to pass an attention check to confirm understanding. Before starting the main task, they underwent a training session where they performed 12 trials using an independent set of stimuli. The main task consisted of 288 trials divided into six blocks of 48 trials each. In between blocks, participants were prompted to take a short break (maximum 2–3 min) to maintain attention and accuracy. Each trial began with a fixation target (Thaler et al., 2013) presented at the center of the screen for a jittered duration between 500 and 1000 ms, which was randomized in 100-ms steps. Then, participants were shown three images: one reference image on top and two probe images below (see Fig. 8). Their task was to judge which of the two probe images was more similar to the reference image. Participants made their choices by pressing the “f” and “j” keys to select, respectively, the left or the right probe. They were asked to respond within 5 s and – if they did not – after their response they were prompted to respond more quickly. The opacity of the selected image was reduced to 0.5 for 1000 ms, giving participants visual feedback that their response had been registered. At the end of the experiment, participants were given the option to complete a debriefing questionnaire about their experience. They were asked for their overall impressions of the study, whether they encountered any technical problems, if they used any specific strategies during the experiment, and for any additional feedback they might have.

#### A.2.4 Experimental control, data quality, and exclusion criteria

A key drawback of online studies compared to on-site experiments is the lack of experimental control, but there are several strategies to maintain data quality (Rodd, 2024). We implemented both experimental measures during data collection and an analysis pipeline with sanity checks on the collected data. In order to ensure a relative consistency in the experimental environment, participants were required to use a desktop device, the Google Chrome web browser with a minimum screen resolution of 800 x 600 pixels and to remain in fullscreen mode throughout the experiment. These requirements were enforced through a preliminary device and browser check before the experiment began and by pausing the experiment if participants exited fullscreen mode. Every interaction with the browser (e.g., if participant exited fullscreen) was recorded, in addition to the time spent in each phase of the task (e.g., how much time they spent on each break). Such data was crucially important to identify and potentially exclude participants who showed unreliable engagement with the experiment. Participants who did not finish the experiment were also excluded. In addition to the interaction data, we used two behavioral indicators to flag participants with outlying performance: reaction times and response biases. We first examined the distribution of RTs (Fig. 9) and identified these outliers with very short RTs. Any participant with mean RTs below 1000 ms was excluded, assuming that a mean RT below 1000 ms throughout the task would indicate that a participant might have performed the task inattentively. We also examined the distribution of response biases (Fig. 10), making the assumption that participants who pressed one of the two keys with high frequency were not fully engaging with the task. We first calculated the proportion of choices for ‘f’ (left probe) and ‘j’ (right probe) key presses for each participant, to then exclude participants with biases falling 1.85 times outside the interquartile range bounds. Finally, we conducted an analysis of participant feedback provided at the end of the experiment to identify potential technical problems or other issues encountered during the study.

Of 1253 participants who participated in the experiment, 140 (11%) were excluded either because they did not complete all trials (92 participants, 7.3%) or because they did not satisfy the inclusion criteria based on reaction times (36 participants, 2.9%) and response biases (20 participants, 1.6%), with four (0.3%) participants satisfying more than one exclusion criterion. A final sample of 1113 participants passed the criteria and was used for the analysis. The final sample had an average age of 28.9 years (SD = 5.7). The majority of participants were male, accounting for 60% of the sample, while 39.6% were female. A small proportion, 0.4%, chose not to disclose their gender. Each participant provided judgements only for two objects and each object was assigned to at least 20 participants. The majority of objects (56) were judged by 20 participants, 38 objects were judged by 21 participants, and 14 objects were judged by 22 participants (Fig. 11 and Table 2). Because of the very small difference, we included all data in the final analysis.

**Table 2:** Number of participants assigned to each object

Object name	Number of participants assigned
acacia	20
adobe_house	20
bamboo_house	21
baobab	20
barn	21
basket	21
beaver	21
bell_tower	21
birch	22
bottle	21
bristlecone	20
butterfly	21
cabin	20
cactus	21
campervan	22
candlestick	20
car_vintage	21
cat	20
chair	21
cherry	20
coffee_mug	21
desert_arch	22
desert_butte	20
dog	21
dragon_tree	20
elephant	20
eucalyptus	20
farm	21
fish	20
fishing_boat	20
fjord_pillar	20
forest_boulder	20
forest_river	20
fortress	20
frog	20
futuristic_building	20
geothermal_spring	22
giraffe	20
grassland_monolith	21
greenhouse	20
hill_river	22
horse	22
hot_air_balloon	20
hourglass	22
igloo	21
jar	20
jet_plane	20
kapok	22
koala	21
lake_island	22
lamp_standing	21
lamp_table	21
lantern	21
leopard	20
lighthouse	21
lizard	20
lorry	20
magnolia	20
maple	20
modern_building	21
moorland_tor	22
mortar	20
motorcycle	20
mountain_glacier	22
mountain_ridge	21
mountain_rock	20
oak	20
observatory	20
offroad_vehicle	20
olive	21
owl	21
pagoda	20
palm	21
parrot	21
passenger_train	20
penguin	20
pine	20
pine_med	20
polar_base	21
polar_iceberg	20
pot	21
rowing_boat	21
sailboat	21
scooter	21
sea_stack	21
seal	20
seaplane	22
sequoia	20
shovel	20
skyscraper	22
sofa	20
space_rocket	21
sports_car	20
steam_locomotive	20
tea_house	20
teapot	20
telescope	20
tortoise	20
tourist_helicopter	21
tractor	20
tropical_bird	20
tropical_karst	21
vase	22
volcanic_peak	21
wetland_tufa	20
wheelbarrow	20
willow	21
windmill	21

#### A.2.5 Similarity judgement analysis

The main goal of the similarity judgement analysis was to estimate a perceptual scale for each object and its variations, given a set of perceptual judgements. To analyze the triplet judgements, we used three algorithms: maximum likelihood difference scaling (MLDS) (Maloney & Yang, 2003), which is a well-established scaling method in psychophysics, and two ordinal embedding algorithms, soft ordinal embedding (SOE) (Terada & Von Luxburg, 2014) and *t*-distributed stochastic triplet embedding (*t*-STE) (Van Der Maaten & Weinberger, 2012). MLDS can be used only in one-dimensional cases, whereas *t*-STE and SOE have been proposed to find ordinal embeddings in higher-dimensional spaces (Haghiri et al., 2020). Embeddings, in this context, are Euclidean representations that preserve the ordinal relationships among data points based on a set of perceptual triplet judgements (Agarwal et al., 2007; Jamieson & Nowak, 2011). Given a set of triplet constraints $\mathcal {T} = \{ (i, j, k) \}$, derived from perceptual judgements where each triplet (i,j,k) indicates that image i is perceived as more similar to image j than to image k, an embedding is a set of points $\{ x_i \in \mathbb {R}^d \}$, where d denotes the dimensionality of the embedding space, such that:

$$ \Vert x_i - x_j \Vert < \Vert x_i - x_k \Vert , \quad \forall \, (i, j, k) \in \mathcal {T}. $$

Because perceptual judgements are noisy and have inconsistencies, it may not be possible to satisfy all triplet constraints at the same time. For this reason, the embedding $\{ x_i \in \mathbb {R}^d \}$ is obtained by solving an optimization problem that minimizes a loss function over the set of triplet constraints (Agarwal et al., 2007; Jamieson & Nowak, 2011).

We performed a comparative analysis between the three algorithms, assessing both their performance and the stability of the embedding estimates. To assess their performance, we used the cross-validated triplet error $$E_t$$ (Haghiri et al., 2020). Given a set of embedding representations $$ y_1, \ldots , y_n $$ estimated on a triplet set T, the triplet error measures how many triplets from a validation set $T^{\prime }$ are not correctly represented by those embeddings. It is defined as follows (Haghiri et al., 2020):

$$ E_t \!=\! \frac{1}{|T'|} \sum _{t=(i,j,k) \in T'} 1\!\!1\{R_t \cdot \text {sgn}(\Vert \hat{y}_i \!-\! \hat{y}_k\Vert ^2 \!-\! \Vert \hat{y}_i - \hat{y}_j\Vert ^2) \!=\! 1\}, $$

where $$ 1\!\!1 $$ is the indicator function that equals 1 if the given triplet does not satisfy the expected distances and equals 0 if the relationship is satisfied. The triplet error thus counts the number of violations where the distance between the reference point i and point k is less than or equal to the distance between the reference point i and point j. We used a leave-one-participant-out cross-validation to evaluate embeddings for each object using the three different estimators. First, the triplet judgement data were grouped by object. For each object group, we divided the triplet judgements into training and test data. In each fold of the cross-validation, data from one participant were left out as the test set $T^{\prime }$, while the data from the remaining participants constituted the training set T. This ensured that the model was evaluated on data from a participant it had not seen during training, providing an unbiased estimate of its performance. Moreover, it also provided information about how generalizable the embedding estimates were across participants. For each fold of the cross-validation, we ran ten iterations of the embedding estimation procedure for the *t*-STE and SOE algorithms. These methods, which rely on stochastic optimization techniques like gradient descent, are prone to converging on local optima. To mitigate this, we ran the algorithm with multiple initializations and selected the embedding solutions that best fit the data, using the triplet error (calculated on the training set only) as the metric (Haghiri et al., 2020). This approach increases the likelihood of identifying a more globally optimal solution by sampling various potential embeddings and reducing the impact of any single suboptimal result. Unlike *t*-STE and SOE, which often require multiple runs to achieve stable results, MLDS uses a maximum likelihood estimation approach that does not require multiple independent runs. Importantly, in each iteration, the training data was used both to compute the embeddings and to calculate the triplet error to prevent data leakage. After running ten iterations, we selected the embedding with the lowest triplet error as the best fit.

When the embeddings are computed, the scale and orientation of the solutions can vary across different iterations, resulting in inconsistencies such as varying scales and mirror images. To achieve a consistent scale and orientation, the embedding values were subjected to two transformations. First, we normalized them to the [0,1] interval. Second, we standardized the direction of the embeddings by enforcing a descending order using the Theil-Sen estimator (Theil, 1950; Sen, 1968). The estimator fits a line by calculating the median slope from all pairwise slopes of the data points within a sample, providing a robust measure of the general trend of the data. If the slope of the fitted line was positive, indicating an ascending order, we flipped and translated the embeddings (1-y) to achieve the desired descending orientation within the [0,1] interval. Importantly, this method does not affect the triplet error calculation, since the relative distances are preserved. This method is suitable for our use case, which involves aligning the embeddings on a one-dimensional scale. For multidimensional embeddings, generalized Procrustes analysis or unsupervised methods (Gower, 1975; Grave et al., 2019).

For each object and category, we calculated the mean cross-validated triplet error of each algorithm by averaging the triplet errors from all cross-validation steps. The object-wise and category-wise errors estimated how well the model performed for individual objects and for categories. We also calculated the overall mean triplet error for each algorithm, which provided a general measure of how they performed across objects.

As a final step, we reordered the images according to the embedding results and compared this order to the one defined by the LPIPS metric.

#### A.2.6 Physical characteristics of the stimuli set

We calculated the physical characteristics of the stimulus set^6^. These measures were calculated only for the anchor images. When necessary, color images were converted to grayscale using the OpenCV *cvtColor* function with the *COLOR_RGB2GRAY* conversion code (Bradski, 2000). This function implements a color space conversion applying the following weighted sum of the RGB channels (OpenCV, 2024):

$$ \text {gray\_image} = 0.299 \cdot R + 0.587 \cdot G + 0.114 \cdot B $$

We calculated the following measures:

- **Contrast:** Defined as the standard deviation of the pixel intensity values in the normalized grayscale image. This measure captures the variation in intensity within the image and indicates the overall variability in brightness:
  $$ \text {contrast} = \sqrt{\frac{1}{N} \sum _{i=1}^{N} \left( \frac{\text {gray\_image}(i)}{255.0} - \mu \right) ^2} $$
  where N is the total number of pixels, $\text {gray\_image}(i)$ is the intensity of the i-th pixel, and μ is the mean intensity of the normalized grayscale image:
  $$ \mu = \frac{1}{N} \sum _{i=1}^{N} \frac{\text {gray\_image}(i)}{255.0} $$
  Normalizing by 255, which is the maximum intensity value for an 8-bit grayscale image, scales the pixel intensity values to a range between 0 and 1 and allows for consistent contrast evaluation across different images.
- **Mean intensity:** Calculated as the mean pixel intensity of the normalized grayscale image. The luminance was calculated using the formula:
  $$ \text {mean\_intensity} = \frac{1}{N} \sum _{i=1}^{N} \text {gray\_image}(i) $$
  where N is the total number of pixels and $\text {gray\_image}(i)$ is the intensity of the i-th pixel.
- **Relative luminance:** Relative luminance takes into account the perception of brightness by considering the different sensitivities of the human eye to red, green, and blue light (BT, 2002). Calculated by first converting gamma-compressed RGB values to linear RGB values using a 2.2 power curve:
  $$ R_{\text {lin}} = R'^{2.2}, \quad G_{\text {lin}} = G'^{2.2}, \quad B_{\text {lin}} = B'^{2.2} $$
  Then applying the relative luminance formula according to the BT.709 standard (BT, 2002):
  $$ Y = 0.2126 \cdot R_{\text {lin}} + 0.7152 \cdot G_{\text {lin}} + 0.0722 \cdot B_{\text {lin}} $$
- **Colorfulness:** The method for calculating image colorfulness is based on the approach by Hasler and Süsstrunk (Hasler & Süsstrunk, 2003). This method uses an opponent color space to quantify colorfulness and they show it correlates with human perceptual judgements. Two channels, rg and yb, represent the differences between the red and green channels, and a combination of the red, green, and blue channels, respectively. These channels are computed as follows:
  rg=R-G
  yb=0.5·(R+G)-B
  The mean (μ) and standard deviation (σ) for these channels are calculated as:
  $$ \sigma _{rgyb} = \sqrt{\sigma _{rg}^2 + \sigma _{yb}^2} $$
  $$ \mu _{rgyb} = \sqrt{\mu _{rg}^2 + \mu _{yb}^2} $$
  The colorfulness metric is then defined as:
  $$ \hat{M}(3) = \sigma _{rgyb} + 0.3 \cdot \mu _{rgyb} $$
  Here, $\sigma _{rgyb}$ combines the variability of the rg and yb channels, while $\mu _{rgyb}$ represents their combined mean value, adjusted by a factor of 0.3.
- **Shannon entropy:** Entropy measures the amount of information or randomness in an image by analyzing the distribution of pixel intensity values. Higher entropy indicates more complexity and variation in pixel intensity values and reflects the richness of detail in the image. It was computed using Shannon’s formula (Shannon, 1948):
  $$ S = -\sum p_k \log (p_k), $$
  where $p_k$ represents the probability of a pixel having intensity value k, calculated from the frequency of each intensity value. S quantifies how unpredictable the pixel intensities are within the image. This calculation was performed using the *skimage.measure.shannon_entropy* function from the *scikit-image 0.24.0* package.
- **Edge density:** Edge density is defined as the proportion of edge pixels to the number of pixels in a portion of the image (Phung & Bouzerdoum, 2007). This measure indicates the amount of structural detail present, with higher edge density suggesting more edges and thus more complexity (Rosenholtz et al., 2007). Edge density was calculated using the Canny edge detection algorithm (Canny, 1986) and considering all pixels within the image. The Canny algorithm identifies areas of steep intensity change through a multi-stage process and outputs a binary map where edge pixels are marked. The formula for edge density is given by:
  $$ \text {edge\_density} = \frac{N_{\text {edge}}}{W \times H} $$
  - $N_{\text {edge}}$ is the number of edge pixels identified by the Canny edge detection algorithm.
  - W is the width of the image.
  - H is the height of the image.
  The algorithm was implemented using the *feature.canny* function from *scikit-image* 0.24.0.
- **Blur strength:** Blur strength estimates the amount of blurring present in an image. It was calculated using the *skimage.measure.blur_effect* function, based on the method described by Crété-Roffet et al. (2007), which developed a perceptual blur metric that correlates well with human perception of blur and has been validated through subjective tests.
- **Sharpness:** Sharpness measures the clarity of edges and fine details in an image. To quantify sharpness, we follow the method outlined by Pech-Pacheco et al. (2000), which relies on a Laplacian operator detecting regions of rapid intensity change (edges) by computing the second spatial derivative of the image. The sharpness metric, $\text {LAP\_VAR}(I)$, is defined as the variance of the Laplacian values. First, a Laplacian mask is applied to the image to compute the Laplacian values L(m,n) at each pixel (m,n). The mask is:
  $$ \text {laplacian\_mask} = \begin{bmatrix} 0 & -1 & 0 \\ -1 & 4 & -1 \\ 0 & -1 & 0 \end{bmatrix} $$
  This operation enhances the edges by emphasizing regions where there is a significant change in intensity. Next, the mean of these absolute Laplacian values, $\bar{L}$, is calculated as follows:
  $$ \bar{L} = \frac{1}{NM} \sum _{m=1}^{M} \sum _{n=1}^{N} |L(m,n)| $$
  where M and N are the dimensions of the image, and |L(m,n)| represents the absolute value of the Laplacian at pixel (m,n). The sharpness metric, $\text {LAP\_VAR}(I)$, is then computed as the variance of these Laplacian values around the mean:
  $$ \text {LAP\_VAR}(I) = \sum _{m=1}^{M} \sum _{n=1}^{N} \left( |L(m,n)| - \bar{L} \right) ^2 $$
  In this formula:
  - L(m,n) is the Laplacian value at the pixel (m,n).
  - $\bar{L}$ is the mean of the absolute Laplacian values.
  High variance in these values indicates a sharp image with well-defined edges, whereas low variance suggests a blurred image with less distinct edges.
- **Spectral energy:** Image statistics vary depending on scene content and viewing conditions (Torralba & Oliva, 2003). Second-order statistics such as the power spectrum, which describe the relationships between pixel intensities, are strongly correlated with scene scale (the spatial extent and distance of scene elements) and scene category (e.g., natural or man-made environments). Analyzing the spectral energy of images helps in understanding low-level features of the stimuli (Cooper et al., 2023). Using the Natural Image Statistical Toolbox for MATLAB (Bainbridge & Oliva, 2015), adapted for Python, we measured the spectral characteristics of each image. We determined the dominant spatial frequencies by finding the frequency below which 80% of the total spectral energy is contained. We also assessed high spatial frequencies by calculating the proportion of frequencies above ten cycles per image. Such a metric quantifies the amount of fine details and textures within the image.

#### A.2.7 Programming tools

The experiment was implemented using the jsPsych library 7.3.1 (Leeuw et al., 2023) and JATOS (Lange et al., 2015), which was installed on a server at the Bernstein Center for Computational Neuroscience (Berlin, Germany). Experimental condition assignments were managed via an external database^7^ and a backend application using Python FastAPI 0.111.0, hosted on Vercel^8^. To run the embedding analyses, we used the Python package *cblearn* (Künstle & Luxburg, 2024), as well as the original MATLAB code for *t*-STE^9^ and the original R code for MLDS (Knoblauch & Maloney, 2008) for replication tests. Since we could successfully replicate the results, we report only the final results obtained using the *cblearn* package.

### A.3 Memory experiment

#### A.3.1 Experimental control, data quality, and exclusion criteria

To ensure high data quality, we applied similar experimental measures as in the Perceptual Similarity Judgement Task (see A.2.4). Participants were required to use a desktop device, Google Chrome as the web browser, a minimum screen resolution of 800×600 pixels, and fullscreen mode throughout the task. A preliminary device and browser check ensured that these requirements were met before the experiment began. If participants exited fullscreen mode during the task, the experiment was automatically paused. Participants were excluded if they did not finish the experiment (12.43%), encountered technical issues (8.88%), or had a mean reaction time below 500 ms or an extreme response bias (1.85 times outside the interquartile range bounds)(3.85%). Mean reaction time and response bias were used as indicators of participants who might have engaged poorly with the task (e.g., repeatedly pressing the same key without performing the task). In addition, we also excluded participants with very poor behavioral performance (17.16%), quantified as a mean accuracy below chance level (50%).

#### A.3.2 Selection of the targets and foils

First, we computed the absolute differences between consecutive embedding values for each object’s set of images. Next, we set a threshold at the 90th percentile of these differences. Consecutive pairs exceeding this threshold were considered discontinuities. If a discontinuity occurred at the beginning or end of a sequence, we marked the corresponding images as outliers. We then selected the target image. This was the image with the highest embedding value that was not marked as an outlier and was not immediately followed by a discontinuity. This procedure made sure that the next image, the “Hard” foil, was perceptually similar to the target. After choosing the target and removing outliers, we divided the remaining images into three bins. The bins corresponded to the 25th, 50th, and 75th percentile of embedding values. From each bin, we selected one foil image whose embedding value was closest to the bin’s percentile.

#### A.3.3 Model specification

The primary goal of our analysis was to examine how learning progressed over time, measured as changes in accuracy across experimental blocks, and to determine whether these learning trajectories differed depending on two primary experimental manipulations: target condition and difficulty. The target condition was modeled as a two-level factor (repeated versus non-repeated targets), difficulty as a three-level factor (easy, medium, hard), and block as a continuous variable ranging from 1 to 6. The fixed effects accounted for the population-level effects of block, difficulty, target condition, for all two-way interactions (block × difficulty, block × target condition, difficulty × target condition), and for the three-way interaction (block × difficulty × target condition). The random effects structure accounted for participant-specific variability through random intercepts, representing individual baseline performance levels, and random slopes for block, reflecting individual differences in learning rates over time.

For each trial *i* of participant *j*, the binary accuracy outcome $y_{ij}$ is modeled as a Bernoulli-distributed random variable:

1 $$\begin{aligned} y_{ij} \sim \text {Bernoulli}(p_{ij}) \end{aligned}$$

The probability of a correct response $p_{ij}$ is linked to a linear predictor via the logit function:

2 $$\begin{aligned} \begin{aligned} \text {logit}(p_{ij}) =\;&\beta _0 + \beta _1 \cdot \text {block}_{ij} \\&+ \beta _2 \cdot \text {difficulty\_medium}_{ij} + \beta _3 \cdot \text {difficulty\_hard}_{ij} \\&+ \beta _4 \cdot \text {target\_condition}_{ij} \\&+ \beta _5 \cdot (\text {block}_{ij} \times \text {difficulty\_medium}_{ij}) + \beta _6 \cdot (\text {block}_{ij} \times \text {difficulty\_hard}_{ij}) \\&+ \beta _7 \cdot (\text {block}_{ij} \times \text {target\_condition}_{ij}) \\&+ \beta _8 \cdot (\text {difficulty\_medium}_{ij} \times \text {target\_condition}_{ij}) + \beta _9 \cdot (\text {difficulty\_hard}_{ij} \times \text {target\_condition}_{ij}) \\&+ \beta _{10} \cdot (\text {block}_{ij} \times \text {difficulty\_medium}_{ij} \times \text {target\_condition}_{ij}) \\&+ \beta _{11} \cdot (\text {block}_{ij} \times \text {difficulty\_hard}_{ij} \times \text {target\_condition}_{ij}) \\&+ u_{0j} + u_{1j} \cdot \text {block}_{ij}. \end{aligned} \end{aligned}$$

where $\beta _0$ represents the baseline log-odds of a correct response under the reference conditions (e.g., initial block, Easy difficulty, non-repeated stimuli). The coefficients $\beta _1$ through $\beta _{11}$ model the fixed effects of the predictors and their interactions. To account for individual differences among participants, the model incorporates random effects for both the intercept and the slope of the block variable:

3 $$\begin{aligned} \begin{pmatrix} u_{0j} \\ u_{1j} \end{pmatrix} \sim \mathcal {N} \left( \begin{pmatrix} 0 \\ 0 \end{pmatrix}, \mathbf {\Sigma } \right) \end{aligned}$$

where $u_{0j}$ is the random intercept (modeling participant-specific baseline performance) and $u_{1j}$ is the random slope for the block (modeling participant-specific learning rates).

The random intercept ($u_{0j}$) and random slope ($u_{1j}$) were allowed to correlate, with covariance matrix:

4 $$\begin{aligned} \mathbf {\Sigma } = \begin{bmatrix} \sigma _{u_0}^2 & \rho \sigma _{u_0} \sigma _{u_1} \\ \rho \sigma _{u_0} \sigma _{u_1} & \sigma _{u_1}^2 \end{bmatrix} \end{aligned}$$

where $\sigma _{u_0}$ and $\sigma _{u_1}$ are the standard deviations of the random intercept and slope, respectively, and ρ represents their correlation.

The prior distributions for the model parameters were selected to be weakly informative to help stabilize parameter estimates (Gelman, 2006). Fixed effects were assigned normal priors:

5 $$\begin{aligned} \beta _k \sim \mathcal {N}(0, \sigma ^2), \quad k = 0,1,\dots ,11 \end{aligned}$$

with $\sigma ^2 = 4$, constraining coefficients to plausible ranges on the log-odds scale (±4 log-odds at 2SD) and centering the effect at 0. Random effect standard deviations ($\sigma _{u0}, \sigma _{u1}$) were assigned:

6 $$\begin{aligned} \sigma _u \sim \text {Half-Cauchy}(0, 1) \end{aligned}$$

priors, which ensure positive values and favor smaller variability across participants while still permitting larger deviations (Gelman et al., 1995; Polson & Scott, 2012).

The prior for the correlation between random effects (ρ) was modeled using a Lewandowski–Kurowicka–Joe (LKJ) distribution (Lewandowski et al., 2009):

7 $$\begin{aligned} \rho \sim \text {LKJ}(\zeta ) \end{aligned}$$

with ζ=2 as a weakly informative prior that discourages extreme correlations, unless supported by the data (Bürkner, 2017). These priors were evaluated through prior predictive checks to confirm they generated reasonable simulated data. In addition, we analyzed other weakly informative priors to assess if they would produce similar results. Posterior predictive checks were conducted to evaluate model fit.

## Appendix B: Extended results

### B.1 Image generation

#### B.1.1 Exemplar images

We generated 60 exemplar images for each of the 108 objects, for a total of 6480 images. The synthesized images were visually evaluated for their quality and fidelity to the input textual prompts, which was generally high. The application of the LoRA model visibly enhanced the quality of the generated images, especially making contrast and scene lighting more realistic.

After visual inspection, 215/6480 images (3.3%) were excluded (Fig. 13). We provide some examples of images that we excluded, highlighting their artefacts in Fig. 12. The distribution of these exclusions by category showed variability in exclusion rates across different categories: the animal category had the highest number of exclusions, with 73 images excluded, accounting for approximately 34% of the total exclusions. The item category followed with 55 excluded images, making up about 26% of the exclusions. The vehicle category had 54 exclusions, representing 25% of the total. The plant category saw 17 images excluded, which is roughly 8% of the total. The landscape element category had 16 exclusions, constituting about 7% of the total. Notably, the building category had no exclusions. An object-specific breakdown (Table 3) shows that while some objects faced high exclusion rates (e.g., 30% of the giraffe images presented visible artefacts), 79/108 (73.1%) objects had fewer than three exclusions, of which 57/108 objects (52.8%) had no exclusions at all.

**Table 3:** Exclusion rates for all exemplar images

	Object	Total_excluded	Total_images	Exclusion_rate
0	windmill	0	60	0.000000
1	polar_iceberg	0	60	0.000000
2	grassland_monolith	0	60	0.000000
3	greenhouse	0	60	0.000000
4	hill_river	0	60	0.000000
5	hot_air_balloon	0	60	0.000000
6	polar_base	0	60	0.000000
7	igloo	0	60	0.000000
8	pine_med	0	60	0.000000
9	kapok	0	60	0.000000
10	pine	0	60	0.000000
11	lake_island	0	60	0.000000
12	lamp_table	0	60	0.000000
13	lantern	0	60	0.000000
14	willow	0	60	0.000000
15	lighthouse	0	60	0.000000
16	lorry	0	60	0.000000
17	magnolia	0	60	0.000000
18	maple	0	60	0.000000
19	modern_building	0	60	0.000000
20	moorland_tor	0	60	0.000000
21	palm	0	60	0.000000
22	motorcycle	0	60	0.000000
23	mountain_glacier	0	60	0.000000
24	mountain_ridge	0	60	0.000000
25	mountain_rock	0	60	0.000000
26	oak	0	60	0.000000
27	observatory	0	60	0.000000
28	offroad_vehicle	0	60	0.000000
29	olive	0	60	0.000000
30	geothermal_spring	0	60	0.000000
31	fortress	0	60	0.000000
32	futuristic_building	0	60	0.000000
33	forest_boulder	0	60	0.000000
34	adobe_house	0	60	0.000000
35	bamboo_house	0	60	0.000000
36	wetland_tufa	0	60	0.000000
37	barn	0	60	0.000000
38	volcanic_peak	0	60	0.000000
39	vase	0	60	0.000000
40	bell_tower	0	60	0.000000
41	birch	0	60	0.000000
42	bottle	0	60	0.000000
43	bristlecone	0	60	0.000000
44	cabin	0	60	0.000000
45	car_vintage	0	60	0.000000
46	forest_river	0	60	0.000000
47	tea_house	0	60	0.000000
48	steam_locomotive	0	60	0.000000
49	teapot	0	60	0.000000
50	dragon_tree	0	60	0.000000
51	fishing_boat	0	60	0.000000
52	farm	0	60	0.000000
53	scooter	0	60	0.000000
54	sea_stack	0	60	0.000000
55	sofa	0	60	0.000000
56	pagoda	0	60	0.000000
57	skyscraper	0	60	0.000000
58	cactus	1	60	0.016667
59	shovel	1	60	0.016667
60	tropical_karst	1	60	0.016667
61	tropical_bird	1	60	0.016667
62	horse	1	60	0.016667
63	jar	1	60	0.016667
64	fish	1	60	0.016667
65	desert_arch	1	60	0.016667
66	sailboat	1	60	0.016667
67	owl	1	60	0.016667
68	frog	1	60	0.016667
69	campervan	1	60	0.016667
70	penguin	1	60	0.016667
71	parrot	2	60	0.033333
72	acacia	2	60	0.033333
73	lizard	2	60	0.033333
74	baobab	2	60	0.033333
75	candlestick	2	60	0.033333
76	eucalyptus	2	60	0.033333
77	cat	2	60	0.033333
78	dog	2	60	0.033333
79	beaver	3	60	0.050000
80	hourglass	3	60	0.050000
81	tractor	3	60	0.050000
82	pot	3	60	0.050000
83	tortoise	3	60	0.050000
84	cherry	3	60	0.050000
85	desert_butte	3	60	0.050000
86	butterfly	4	60	0.066667
87	telescope	4	60	0.066667
88	coffee_mug	4	60	0.066667
89	jet_plane	4	60	0.066667
90	elephant	4	60	0.066667
91	tourist_helicopter	5	60	0.083333
92	basket	5	60	0.083333
93	chair	5	60	0.083333
94	passenger_train	6	60	0.100000
95	leopard	6	60	0.100000
96	sequoia	7	60	0.116667
97	lamp_standing	7	60	0.116667
98	sports_car	8	60	0.133333
99	space_rocket	8	60	0.133333
100	rowing_boat	9	60	0.150000
101	wheelbarrow	9	60	0.150000
102	seal	9	60	0.150000
103	seaplane	9	60	0.150000
104	fjord_pillar	11	60	0.183333
105	mortar	11	60	0.183333
106	koala	12	60	0.200000
107	giraffe	18	60	0.300000

#### B.1.2 Pair selection using the LPIPS metric

For each object, we calculated the LPIPS scores between all pairs of the exemplar images that had not been excluded (Fig. 14). Overall, the mean LPIPS score across all objects was 0.453 (SD=0.054). The object with the highest mean LPIPS score was fish (M=0.589, SD=0.039) and the one with the lowest was birch (M=0.286, SD=0.344). We then examined the distribution of mean LPIPS scores for each category. The category items had the highest mean LPIPS score (M=0.483, SD=0.055), followed by animal (M=0.469, SD=0.068), vehicle (M=0.450, SD=0.028), landscape element (M=0.446, SD=0.054), plant (M=0.442, SD=0.055), and building had the lowest mean score (M=0.430, SD=0.043). A one-way analysis of variance (ANOVA) revealed a statistically significant difference in mean LPIPS scores by category (F(5,24)=2.54, p=0.033). To investigate the pairwise differences more in detail, we conducted a post hoc Tukey HSD test (Tukey, 1949), which indicated a significant difference in mean LPIPS scores between the categories building and item (mean difference =0.0538, p=0.030). No other pairwise comparisons showed statistically significant differences in mean LPIPS scores (p>0.05). All test results are reported in Table 4.

**Table 4:** Multiple comparison of means - Tukey HSD, family-wise error rate (FWER) = 0.05, between LPIPS scores from all non-excluded anchor images

group1	group2	meandiff	p-adj	lower	upper	reject
animal	building	-0.0399	0.2052	-0.0904	0.0106	False
animal	item	0.0139	0.9671	-0.0366	0.0644	False
animal	landscape_element	-0.0238	0.7459	-0.0743	0.0267	False
animal	plant	-0.0274	0.6156	-0.0779	0.0231	False
animal	vehicle	-0.0192	0.8779	-0.0697	0.0313	False
building	item	0.0538	0.0297	0.0033	0.1043	True
building	landscape_element	0.0161	0.9383	-0.0344	0.0666	False
building	plant	0.0125	0.9792	-0.038	0.063	False
building	vehicle	0.0207	0.8406	-0.0298	0.0712	False
item	landscape_element	-0.0377	0.2624	-0.0882	0.0128	False
item	plant	-0.0413	0.1747	-0.0918	0.0092	False
item	vehicle	-0.0331	0.4052	-0.0836	0.0174	False
landscape_element	plant	-0.0036	0.9999	-0.0541	0.0469	False
landscape_element	vehicle	0.0046	0.9998	-0.0459	0.055	False
plant	vehicle	0.0082	0.997	-0.0423	0.0587	False

The table reports pairwise comparisons among different categories with the mean difference, adjusted *p* values, and confidence intervals. Significant differences at the 0.05 level are indicated in the reject column

Following the procedure illustrated in the Methods (see Appendix A.1.3 and  A.1.4), for each object we selected a pair of images (an *anchor image* and a *guide image*) to interpolate. All anchor images are shown in Fig. 2 and  3. The performance of this procedure was assessed through visual inspection. In the vast majority of cases (89.81% of image pairs), our procedure successfully selected a suitable pair of images on the first attempt. However, in 11 cases (10.18%), the initially selected second image did not meet our specified conditions (as detailed in the Methods section), necessitating the use of backup images. Specifically, we resorted to the second choice for seven object pairs, the third choice for three pairs, and the fourth choice for two pairs. We assessed the statistical distribution of LPIPS scores between the selected pairs, that is, the score between the *anchor* and the *guide image* (Fig. 15). The mean LPIPS score was 0.35 (SD=0.067). Pairs from the animal category had a mean score of 0.393 (SD=0.073), from buildings had a mean score of 0.334 (SD=0.052), from items had a mean score of 0.370 (SD=0.087), from landscape elements had a mean score of 0.340 (SD=0.053), from plants had a mean score of 0.335 (SD=0.063), and from vehicles had a mean score of 0.339 (SD=0.052). A one-way ANOVA test indicated a statistically significant difference in mean LPIPS scores across categories (F(5,120)=2.525, p=0.034). However, subsequent pairwise comparisons using the Tukey HSD test did not reveal significant differences between any specific pairs of categories (all p>0.05), see Table 5. This suggests that while pairs from different categories differed in mean LPIPS scores, these differences were relatively subtle.

**Table 5:** Multiple comparison of means - Tukey HSD, FWER = 0.05, between LPIPS scores from selected pairs of *anchor* and *guide* images in the interpolations

Group1	Group2	Meandiff	p-adj	Lower	Upper	Reject
animal	building	-0.0589	0.0791	-0.1216	0.0039	False
animal	item	-0.0225	0.9033	-0.0852	0.0403	False
animal	landscape_element	-0.0533	0.1434	-0.1161	0.0094	False
animal	plant	-0.0577	0.0902	-0.1204	0.0051	False
animal	vehicle	-0.0541	0.1325	-0.1168	0.0086	False
building	item	0.0364	0.5448	-0.0263	0.0991	False
building	landscape_element	0.0055	0.9998	-0.0572	0.0683	False
building	plant	0.0012	1.0	-0.0616	0.0639	False
building	vehicle	0.0048	0.9999	-0.0580	0.0675	False
item	landscape_element	-0.0309	0.7096	-0.0936	0.0319	False
item	plant	-0.0352	0.5803	-0.0980	0.0275	False
item	vehicle	-0.0316	0.6873	-0.0944	0.0311	False
landscape_element	plant	-0.0044	1.0	-0.0671	0.0584	False
landscape_element	vehicle	-0.0008	1.0	-0.0635	0.0620	False
plant	vehicle	0.0036	1.0	-0.0591	0.0663	False

The table presents mean differences, adjusted p-values, and confidence intervals for each pairwise comparison

#### B.1.3 Interpolations

For each of the 108 object pairs, we generated 200 interpolated images (including the *anchor* and the *guide* images). Following the criteria explained in the Methods (Appendix A.1.5), we selected ten images for each object, including the *anchor image* and nine of its interpolations. To achieve this, we first computed the LPIPS score between the *anchor image* and each interpolated image.

Results are shown in Fig. 18. Overall, the mean LPIPS score across all objects was 0.249 (SD=0.055). The object with the highest mean LPIPS score was fish (M=0.401, SD=0.111), and the one with the lowest was birch (M=0.116, SD=0.019). The object with the highest standard deviation in LPIPS scores was a sailboat in the vehicle category (M=0.306, SD=0.138), whereas the object with the lowest standard deviation in LPIPS scores was birch in the plant category (M=0.116, SD=0.019). We then examined the mean LPIPS scores for each category. Animal had a mean LPIPS score (M=0.277, SD=0.053), followed by item (M=0.264, SD=0.069), landscape element (M=0.238, SD=0.045), plant (M=0.238, SD=0.055), building (M=0.237, SD=0.042), and vehicle (M=0.237, SD=0.053). A one-way ANOVA revealed no statistically significant difference in mean LPIPS scores across categories (F(5,24)=1.919, p=0.098).

We defined target similarity scores that were linearly spaced between the minimum and maximum LPIPS values observed for each object. For each target score, we selected the image whose similarity score was closest to the target score, ensuring no duplicates. We visually inspected the interpolated images to exclude those with artefacts and maintain a high level of quality and consistency. Given the vast number of images (21,600 in total), the visual inspection was performed only after selecting the interpolated images of interest. For each object, we assessed how many interpolations needed to be swapped with one of their neighbors. The vast majority of objects (87.62%) required no swaps, while a total of 16 objects (14.81%) required one or more swaps. Among those, 8 were from *animal* (44.4% of the objects from that category), 4 from *item* (22.2%), 3 from *vehicle* (16.7%), and 1 from *landscape element* (5.5%). See Fig. 16.

We performed a Pearson correlation to assess the relationship between exclusion rates in anchor images and swap rates in interpolated images. The analysis revealed a significant positive correlation (r=0.531, p<0.001), indicating that objects with higher exclusion rates in exemplar images also tended to have higher swap rates in interpolated images, suggesting consistent issues with the generation of certain objects (Fig. 17). The most problematic was the *animal* category, with an average exclusion rate of 0.068 and an average swap rate of 0.094. The least problematic categories were *building* and *plant*, both with no exclusions. Additionally, the *building* category had no swaps, while the *plant* category had a minimal swap rate (0.016).

#### B.1.4 Physical characteristics of the stimulus set

Figure 19b shows how image features vary by category. Contrast is higher in *animal* and *building*, with symmetrical distributions. Mean pixel intensity is elevated in *building* and *item*, both normally distributed. Relative luminance is uniform across categories, with a slight increase in *animal*. Colorfulness peaks in *plant* and *landscape element*, with normal distributions. Entropy is consistent, but higher in *item* and *vehicle*, with slight left skew. Edge density is higher in *animal* and *item*, and right-skewed. Blur increases in *building* and *item*, with symmetrical distributions. Sharpness is higher in *animal* and *item*, and right-skewed. Image energy is elevated in *building* and *item*, with right skew. High spatial frequency content is also higher in *building* and *item*, with slight left skew.

### B.2 Similarity judgement task

#### B.2.1 Online task

The median experiment duration was 24.11 min and the median duration of the triplet comparison task (excluding instructions and training) was 20.5 min. The median reaction time in the task was 1501.0 ms (Fig. 20a).

#### B.2.2 Evaluation of the embeddings

The performance of the three embedding algorithms of interest – *t*-STE, SOE, and MLDS – was evaluated using the leave-one-participant-out cross-validated triplet error (see Methods in Appendix A.2.5). In order to assess the overall performance of each model, we calculated the mean cross-validated error by method, averaging across all objects. The mean cross-validated triplet error rates were 0.313 for MLDS, 0.280 for *t*-STE, and 0.279 for SOE (Fig. 21a). A one-way ANOVA revealed a significant effect of the embedding algorithm on the triplet error rate, $F(2, 87) = 114.3, \textit{p} < 0.0001$. Post hoc comparisons using the Tukey HSD test indicated that both *t*-STE and SOE had significantly lower triplet error rates compared to MLDS (p<0.05). Specifically, the mean difference between MLDS and SOE was -0.0335 ($95\% \, \text {CI} = [-0.0395, -0.0276]$), and between MLDS and *t*-STE was -0.0332 ($95\% \, \text {CI} = [-0.0391, -0.0272]$), both significant at $\textit{p} < 0.05$. There was no significant difference between *t*-STE and SOE (mean difference = 0.0004, $95\% \, \text {CI} = [-0.0056, 0.0064]$, $\textit{p} = 0.9872$).

We also assessed object-specific and category-specific performance by aggregating and averaging the cross-validated errors for each object (Fig. 21d) and category (Fig. 21c). Our analysis revealed that *t*-STE and SOE consistently performed well across different objects. In contrast, MLDS showed significant performance variability, with a notable error increase for certain objects. For instance, the triplet error for “moorland tor” was 0.48 with MLDS, compared to 0.304 for SOE and 0.305 for *t*-STE. The analysis of category-specific performance showed that *t*-STE and SOE consistently outperformed MLDS across all categories. Notably, *t*-STE and SOE had very similar performance, with negligible differences in triplet errors.

#### B.2.3 Stability of the embedding values

The cross-validated triplet error provides a performance estimate for each algorithm, indicating how well the estimated embeddings generalize to unseen triplets across participants. Since we were ultimately interested in using the participants’ judgements to order the images along a perceptual scale determined by the embedding values, we assessed their stability. Embedding estimates are only useful for this purpose if they are reasonably coherent across the sample and not overly sensitive to variations in the source data. We analyzed the influence of individual participants’ data on the final embedding values by comparing object-specific embedding estimates across folds for each algorithm. In general, we found that SOE provided more stable embedding estimates, making it more robust to variations in the source data. A few object-specific examples are provided in Fig. 22.

#### B.2.4 Reordering the image variations

Although we found no significant difference in performance between SOE and *t*-STE in terms of triplet error, SOE showed more consistent embedding values across iterations, reducing variability in the results (Fig. 22). Therefore, we decided to use the embedding values estimated by SOE to order the image variations on a perceptual scale for each object. The values were computed object-wise, using the aggregate data from all participants. In total, 95 objects (88%) saw at least one position reordering, whereas 13 objects maintained the order provided by the LPIPS score (Fig. 23). The median number of reorderings per object was 3. To quantify the degree of agreement between the ordinal positions derived from LPIPS scores and those from SOE embeddings, we calculated a Spearman’s rank correlation. The analysis revealed a positive correlation, ρ(1078)=0.73, p<0.001, indicating that the ordinal positions based on the LPIPS metric align with those based on the SOE embedding values. We provide some examples of the stimulus set after psychophysical validation in Fig. 5 (main text). For a comparison between the LPIPS-based order and the order after the behavioral task.

#### B.2.5 Embedding analyses

MLDS, *t*-STE, and SOE generally produced comparable results. This corroborates other studies relying on simulations that showed a substantial equivalence in performance for low-dimensional cases (Vankadara et al., 2023). One open issue is to what extent the embedding values reflect real differences in perceptual similarity.

One limitation of using the embedding values to reorder the images on a perceptual scale is that it is not very robust to small variations in embedding due to noise. This is due to the fact that some of the image variations are very similar to one another and have very similar embedding values. In these cases, the ordinal position of the stimuli is susceptible to noise fluctuations. At the same time, it should be noted that not all reorderings are perceptually meaningful. When stimuli have similar embedding values, it means that participants did not perceive significant differences between them. They can therefore be considered a “perceptual cluster”, within which the order of the stimuli is relatively stochastic.

We made the simplifying assumption that each set of image variations can be ranked using a single dimension that follows the LPIPS metric. An open question is what stimulus features within the objects drove the similarity judgements more strongly and future work should address that. However, we found that – in most cases – it was possible to order naturalistic stimuli along one perceptual dimension in good agreement with the LPIPS metric. Our results extend the existing literature showing that the triplet comparison method can be effectively used for naturalistic images, even for perceptually subtle variations.

### B.3 Memory experiment

#### B.3.1 Bayesian model results

Baseline accuracy for easy, non-repeated trials (intercept) was moderately high, with $\hat{\beta _0}=1.52$ and a 95% credible interval $\textrm{CI}_{95}=[1.30, 1.73]$, corresponding to a baseline probability of success of approximately 78%. Participants exhibited progressive learning gains, with a positive block effect, $\hat{\beta _1} = 0.15$, $\textrm{CI}_{95}=[0.09, 0.21]$, indicating a 17% increase in the odds of success per block, $\textrm{OR}= 1.17$. Task difficulty significantly reduced performance, with hard trials ($\hat{\beta _3}= -0.89$, $\textrm{CI}_{95}=[-1.13, -0.64]$, $\textrm{OR}= 0.41$) showing a 59% decrease in odds, and medium trials ($\hat{\beta _2}= -0.52$, $\textrm{CI}_{95}=[-0.78, -0.27]$, $\textrm{OR}= 0.59$) showing a 41% reduction. While stimulus repetition alone showed no reliable main effect ($\hat{\beta _4}= -0.13$, $\textrm{CI}_{95}=[-0.41, 0.16]$), its interaction with block progression demonstrated robust learning improvements (block × repeated: $\hat{\beta _7}= 0.13$, $\textrm{CI}_{95}=[0.05, 0.22]$, $\textrm{OR}= 1.14$ per block). Higher-order interactions involving difficulty levels showed no credible effects, with confidence intervals spanning zero. Individual differences were pronounced: baseline performance varied substantially across participants (random intercept SD=0.72, $\textrm{CI}_{95}=[0.62, 0.83]$), and learning rates exhibited moderate heterogeneity (random block slope SD=0.16, $\textrm{CI}_{95}=[0.13, 0.18]$). A negative correlation between baseline and learning rate (r=-0.54, $\textrm{CI}_{95}=[-0.67, -0.38]$) suggested that participants with higher initial accuracy improved less over time. Bayesian hypothesis tests provided further corroboration of these findings. We summarize these tests using posterior probabilities ($\textrm{pp}$), indicating the probability of the tested hypothesis given the observed data, and evidence ratios ($\textrm{ER}$), quantifying how much more likely the data are under the tested hypothesis compared to the alternative. We observed decisive support for progressive learning across blocks ($H_1$: $\textrm{ER}= \infty$, $\textrm{pp}= 1.00$) and pronounced accuracy reductions under harder task conditions ($H_2$: hard vs. easy, $\textrm{ER}= \infty$, $\textrm{pp}=1.00$; $H_3$: medium vs. easy, $\textrm{ER}=15,999$, $\textrm{pp}=1.00$). While repeated stimuli alone showed no reliable benefit ($H_4$: $\textrm{ER}=0.24$, $\textrm{pp}=0.19$), their interaction with block progression strongly improved learning ($H_5$: $\textrm{ER}=2,132$, $\textrm{pp}=1.00$). For interactions involving difficulty, we found moderate evidence that hard trials attenuated learning gains ($H_6$: $\textrm{ER}=25.8$, $\textrm{pp}=0.96$), though the effect size was small ($\hat{\beta _6}=-0.06$). In contrast, other interactions, including medium-difficulty effects ($H_7$: $\textrm{ER}=4.48$, $\textrm{pp}=0.82$) and three-way combinations ($H_8-H_{11}$: $\textrm{ER} < 4.5$, $\textrm{pp} \le 0.79$) received negligible support, suggesting repetition effects remained stable across difficulty levels.

**Table 6:** Summary of posterior estimates from the Bayesian hierarchical model

	Model estimates			Diagnostics			
Parameter	β	Estimate	Est. Error	$\textrm{CI}_{95}$	$\hat{R}$	Bulk ESS	Tail ESS
Intercept	$\beta _0$	1.516	0.110	[1.303, 1.734]	1.000	9834	16524
Block (learning effect)	$\beta _1$	0.152	0.029	[0.094, 0.209]	1.000	9181	15058
Medium difficulty (vs. easy)	$\beta _2$	-0.521	0.131	[-0.779, -0.266]	1.000	9998	17470
Hard difficulty (vs. easy)	$\beta _3$	-0.886	0.126	[-1.133, -0.639]	1.000	9808	15487
Repeated condition (vs. non-repeated)	$\beta _4$	-0.126	0.144	[-0.412, 0.157]	1.000	8258	14999
Block × medium difficulty	$\beta _5$	-0.032	0.035	[-0.102, 0.038]	1.000	9553	16399
Block × hard difficulty	$\beta _6$	-0.061	0.034	[-0.128, 0.006]	1.000	9325	16067
Block × repeated condition	$\beta _7$	0.134	0.041	[0.054, 0.216]	1.000	8348	15042
Medium difficulty × repeated condition	$\beta _8$	-0.070	0.189	[-0.441, 0.301]	1.000	9071	16165
Hard difficulty × repeated condition	$\beta _9$	0.028	0.182	[-0.327, 0.387]	1.000	8771	15203
Block × medium difficulty × repeated condition	$\beta _{10}$	0.042	0.054	[-0.063, 0.148]	1.000	9145	17181
Block × hard difficulty × repeated condition	$\beta _{11}$	-0.024	0.051	[-0.125, 0.076]	1.000	8634	14974

The table reports posterior means (*Estimate*), standard errors (*Est. Error*), and 95% credible intervals ($\textrm{CI}_{95}$). Model diagnostics include the Gelman–Rubin convergence statistic ($\hat{R}$) and effective sample sizes (Bulk ESS, Tail ESS), indicating sufficient sampling efficiency

*Note.* $\textrm{CI}_{95}$ = 95% credible interval; ESS = effective sample size; $\hat{\textrm{R}}$ = Gelman–Rubin convergence statistic

## Declarations

### Code availability

Custom code will be made available via GitHub upon publication.
